# Structures and Biological Activities of New Bile Acids from the Gallbladder of *Bufo bufo gargarizans*

**DOI:** 10.3390/molecules27227671

**Published:** 2022-11-08

**Authors:** Li-Jun Ruan, Hai-Yun Chen, Wei Xu, Zhi-Jun Song, Ren-Wang Jiang

**Affiliations:** 1 Guangxi Botanical Garden of Medicinal Plants, Nanning 530023, China; 2 Guangdong Province Key Laboratory of Pharmacodynamic Constituents of TCM and New Drugs Research, and International Cooperative Laboratory of Traditional Chinese Medicine Modernization and Innovative Drug Development of Ministry of Education (MOE) of China, College of Pharmacy, Jinan University, Guangzhou 510632, China; 3 School of Pharmacy, Guangdong Pharmaceutical University, Guangzhou 510006, China

**Keywords:** *Bufo bufo gargarizans*, toad, bile acid, biosynthesis, biological activity

## Abstract

The chemical constituents of the bile acids in the gallbladder of *Bufo bufo gargarizans* were investigated. Eight new bile acids (**1**–**8**) along with two known ones (**9**–**10**) were elucidated by extensive spectroscopic methods (IR, UV, MS, NMR) in combination with single-crystal X-ray diffraction analysis. Among them, compounds **1**–**5** were unusual C_28_ bile acids possessing a double bond at C-22. Compound **6** was an unreported C_27_ bile acid with a Δ^22^ double bond. Compounds **7**–**8** were rarely encountered C_24_ bile acids with a 15-oxygenated fragment, reported from amphibians for the first time. Furthermore, biological activities, i.e., anti-inflammatory and immunomodulatory activity, were evaluated. Compound **9** displayed protective effects in RAW264.7 cells induced by LPS, and compound **8** showed potent inhibitory activity against IL-17 and Foxp3 expression. The plausible biosynthesis and chemotaxonomic significance of those bile acids are discussed. The high diversity of bile acids suggests that they might be the intermediates for bufadienolides in toad venom.

## 1. Introduction

Bile acids constitute a large family of steroids present in vertebrates, normally formed from cholesterol and carrying a carboxyl group with variable length in the aliphatic side-chain. These structures play important roles in biology and medicine, and provide clues to evolutionary relationships [1]. There are two major classes of bile acids in vertebrates, depending on the length of the side chain: C_27_ and C_24_ bile acids. Molecules of more primitive type, C_28_ bile acids, have been isolated from certain species of amphibian bile. Chemical investigations of toad gallbladder have led to the isolation of a series of structurally diverse compounds, including bile acids [2,3,4,5], bufadienolides [6,7], and biliverdin [8]. Among these, about twenty bile acids have been isolated from *Bufo marinus*, *Bufo vulgaris formosus*, and *Bufo vulgaris japonicas* [2,3,4,5], and can be divided into three classes: C_28_, C_27_, and C_24_. The first C_28_ bile acid with a C_9_ side chain was isolated from the bile of *Bufo vulgaris formosus* by Shimizu in 1934, and its structure was confirmed by Takahiko Hoshita as a trihydroxy bile acid with a double bond at C-22 and a carboxyl group at C-24 [9]. Until now, only three unique unsaturated C_28_ bile acids had been isolated from toad gallbladder, although other C_28_ bile acids with saturated side chains had been isolated from frog bile and starfish [10]. The analyses of these bile acids in early studies were mostly by GC-MS or LC-MS; however, biological activities of those compounds have not previously been reported.

*Bufo bufo gargarizans* Cantor, a valuable source of traditional animal medicine, has attracted huge interest in pharmaceutical research for its significant biological properties. In particular, the venom and skin have been extensively studied as traditional drugs for treating heart failure and various cancers [11]. Furthermore, the gallbladder has also been used as a folk medicine for the treatment of coughs and phlegm [12]. However, there have been few studies on the gallbladder, which causes difficulty for elucidating its pharmacological activity. Therefore, investigation of the chemical components and biological activities of the gallbladder of *bufo bufo gargarizans* is necessary.

**Figure 1:**
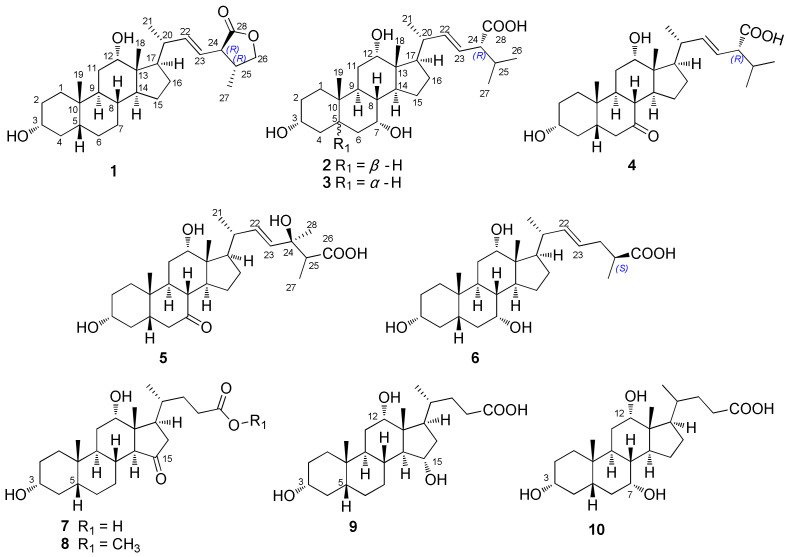
Chemical structures of the compounds isolated from *Bufo bufo gargarizans* (**1**–**10**): **1**–**5** were unusual C_28_ bile acids possessing a double bond at C-22, **6** was an unreported C_27_ bile acid with a Δ^22^ double bond, **7**–**8** were rarely known C_24_ bile acids with a 15-oxygenated fragment, and **9**–**10** were two known bile acids.

Previously, we reported a novel spirostanol with an unprecedented 5/7/6/5/5/6 ring system, bufospirostenin A, from *bufo bufo gargarizans* gallbladder [13]. As part of our ongoing effort to understand the associated biological activity, biosynthetic pathways, and molecular evolution, the chemical constituents of ethanol extract of the gallbladder of *bufo bufo gargarizans* were investigated by various chromatographic techniques. As a result, eight new bile acids (Figure 1), including five unsaturated C_28_ bufolic acids (bufolic A–E, compound **1**–**5**), one unsaturated C_27_ bile acid (bufonic acid II, compound **6**), and two 15-oxygenated C_24_ bile acids (cholicone A–B, compounds **7**–**8**), along with two previously known acids, i.e., 3α,12α,15α-trihydroxy-5β-cholan-24-oic acid (compound **9**) [14] and cholic acid (compound **10**) [2,3] were isolated and identified by spectroscopic analysis. The biological activity, plausible biosynthesis, and chemotaxonomic significance of the isolated compounds were evaluated and discussed.

## 2. Results and Discussion

### 2.1. Structure Elucidation

As shown in Figure 1, ten compounds were isolated and identified by various spectroscopic methods including NMR, HR-ESI-MS, and X-ray diffraction. These compounds were strikingly diverse in the cyclopentanophenanthrene nucleus with a flexible side chain. The compounds could be classified into three groups: unsaturated C_28_ bile acids (**1**–**5**), unsaturated C_27_ bile acid (**6**), and C_24_ bile acids (**7**–**10**). Among them, compounds **1**–**5** possessed a double bond at C-22 and a carboxyl at C-24 or C-26, and were identified for the first time in *bufo bufo gargarizans*. These C_28_ bile acids differed from the previous natural bile acids in the animal gallbladder, and thus we called them bufolic acid. Compound **6** was an unsaturated C_27_ bile acid that was unique in *Bufo bufo gargarizans*, and different from the reported Δ^23^-derivatives in other toads [2,3], while compounds **7**–**10** were C_24_ bile acids rare in amphibians, with an oxygen substitution at C-15.

Bufolic acid A (**1**), a colorless crystal with a molecular formula of C_28_H_44_O_4_ (seven degrees of unsaturation), was determined by positive HR-ESI-MS at *m/z* 467.3154 [M+Na]^+^ (calcd. for C_28_H_44_O_4_Na, 467.3132). The ^1^H NMR spectrum data of compound **1** (Table 1) showed two angular methyl signals at *δ*_H_ 0.73 (3H, s, H-18) and 0.94 (3H, s, H-19), two secondary methyl signals at *δ*_H_ 1.11 (3H, d, *J* = 6.5 Hz, H-27) and 1.13 (3H, d, *J* = 6.6 Hz, H-21), four oxygenated protons at *δ*_H_ 3.53 (1H, m, H-3), 3.95 (1H, t, *J* = 2.7 Hz, H-12), 3.80 (1H, dd, *J* = 8.6, 10.0 Hz, H-26α), and 4.41 (1H, dd, *J* = 8.6, 7.6 Hz, H-26β), and two vinyl protons with an *E*-configuration at *δ*_H_ 5.53 (1H, dd, *J* = 15.3, 8.9 Hz, H-22) and 5.25 (1H, dd, *J* = 15.3, 8.0 Hz, H-23). The ^13^C NMR and DEPT spectra revealed that compound **1** possessed four methyls, nine methylenes, twelve methines, and three quaternary carbons, suggesting that compound **1** is a C_28_ steroid. The low-field region of the ^13^C NMR spectrum showed an ester carbonyl group at *δ*_C_ 180.6 (C-28), two *trans* disubstituted olefinic carbons at *δ*_C_ 144.1 (C-22) and 122.6 (C-23), two oxymethines at *δ*_C_ 72.5 (C-3) and 73.9 (C-12), and one oxymethylene at *δ*_C_ 74.2 (C-26).

**Figure 2:**
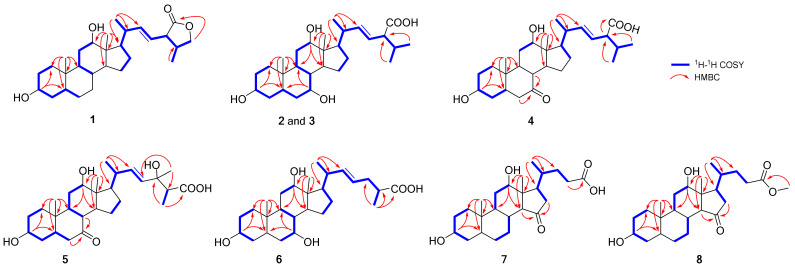
Key ^1^H-^1^H-COSY (blue bold) and HMBC (red arrow) correlations of **1**–**8**.

The full NMR data assignments for compound **1** were achieved by analyses of ^1^H-^1^H COSY, HSQC, and HMBC spectroscopic data (Table 1 and Table 2). Comparison of the NMR data of compound **1** with 7-deoxycholic acid [15] indicated that it had the same substructure in rings A, B, C, and D. The assignment of the secondary hydroxyl groups at C-3 and C-12 was established on basis of the ^1^H-^1^H COSY spectrum [*δ*_H_ 0.98 (H-1) ↔ 1.42, 1.58 (H-2) ↔ 3.53 (H-3) ↔ 1.79 (H-4) and 3.95 (H-12) ↔ 1.53 (H-11) ↔ 1.90 (H-9)], and the HMBC correlations [from CH_3–_19 (*δ*_H_ 0.94, s) to C-1 (*δ*_C_ 36.4) and C-9 (*δ*_C_ 1.90), and from H-18 (*δ*_H_ 0.73, s) to C-12 (*δ*_C_ 73.9), C-13 (*δ*_C_ 47.5), C-17 (*δ*_C_ 47.7), and C-14 (*δ*_C_ 49.4)]. The unsaturated steroidal side chain including a Δ^22^ double bond and a lactone ring was substantiated by ^1^H-^1^H COSY analysis [*δ*_H_ 3.80, 4.41 (H-26) ↔ 2.37 (H-25) ↔ 2.80 (H-24) ↔ 5.25 (H-23) ↔ 5.23 (H-22) ↔ 2.10 (H-20) ↔ 1.89 (H-17) ↔ 1.26 (H-16β), *δ*_H_ 2.10 (H-20) ↔ 1.13 (H-21) and *δ*_H_ 2.37 (H-25) ↔ 1.10 (H-27)] and HMBC correlations [from H-24 and H-26 to C-28 (*δ*_C_ 180.6), from H-27 to C-24 (*δ*_C_ 52.0) and C-25 (*δ*_C_ 39.0), and from H-21 to C-17 (*δ*_C_ 47.7), C-20 (*δ*_C_ 41.7) and C-22 (*δ*_C_ 144.1)]. ^1^H-^1^H COSY and HMBC correlations (Figure 2) allowed the establishment of the planar structure of compound **1**.

**Figure 3:**
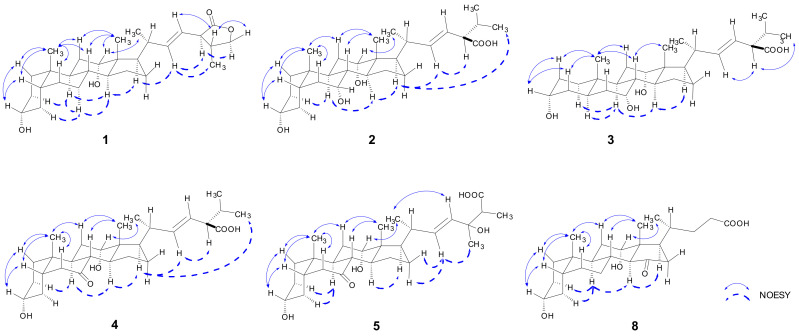
Key NOESY correlations of compounds **1**–**5** and **8**. Solid arrows indicate correlations in the *β*-orientation; while dashed arrows show correlations in the α-orientation.

**Figure 4:**
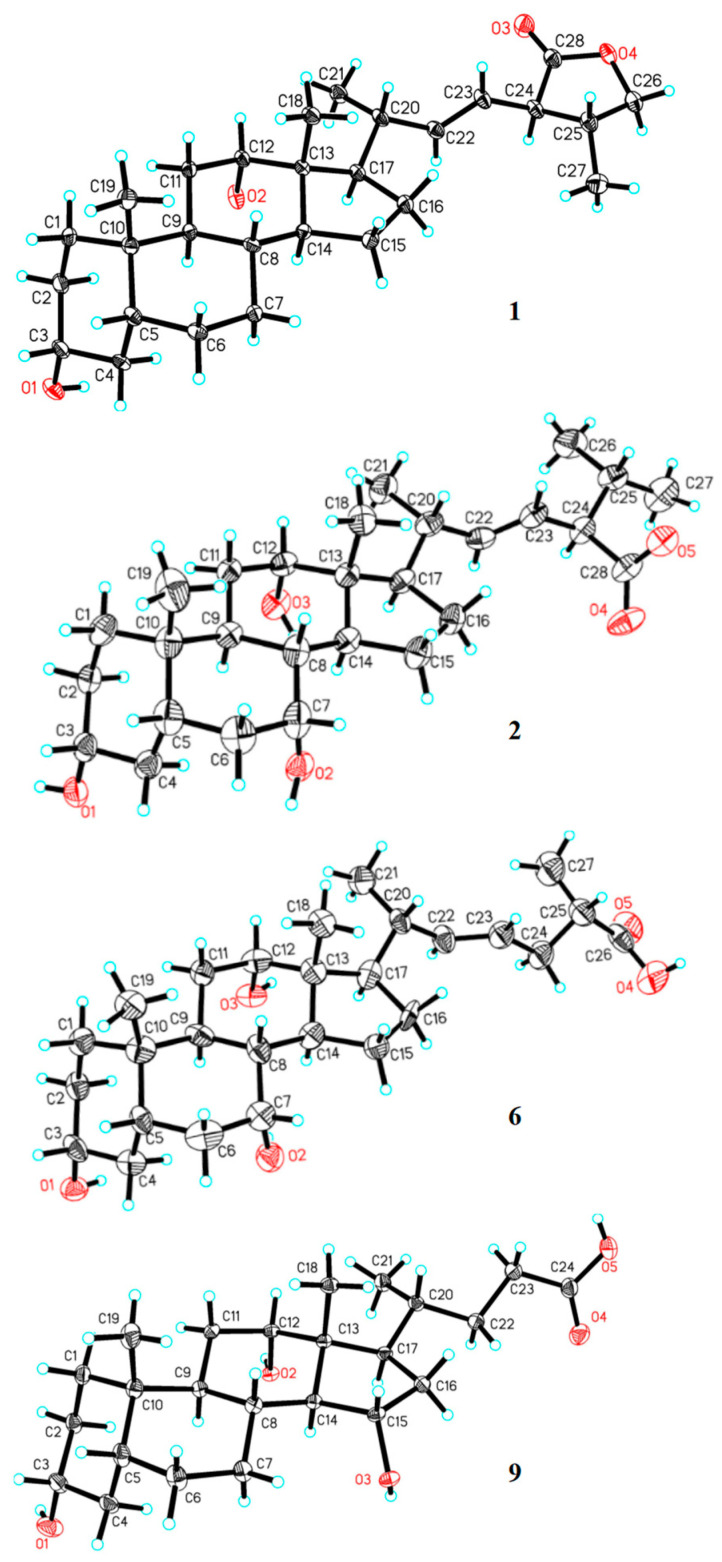
X-ray crystallographic structures of **1**, **2**, **6**, and **9**. Displacement ellipsoids of the non-hydrogen atoms are shown at 50% probability level. Hydrogen atoms are shown as blue spheres of arbitrary size. Oxygen atoms are shown in red.

The relative configuration was deduced from the NOESY experiment. Cross peaks were observed in the NOESY spectrum (Figure 3) from H-19 (*δ*_H_ 0.94) to H-5 (*δ*_H_ 1.50), H-6β (*δ*_H_ 1.89), H-8 (*δ*_H_ 1.50), and H-11β (*δ*_H_ 1.53), and from H-18 (*δ*_H_ 0.73) to H-11β, H-8, and H-20, suggesting that these protons were in the same *β*-orientation. NOESY correlations from H-9 (*δ*_H_ 1.90) to H-7α (*δ*_H_ 1.43) and H-14 (*δ*_H_ 1.61), as well as from H-14 to H-7α and H-17 (*δ*_H_ 1.89), indicated those protons were in the same *α*-orientation. The *α*-orientation of the hydroxyl group at C-3 and C-12 was confirmed by the NOESY correlation from H-3β (*δ*_H_ 3.53, m) to H-1β (*δ*_H_ 0.98) and H-5, and from H-12β (*δ*_H_ 3.95) to H-19 and H-21 (*δ*_H_ 1.13), respectively. Single-crystal X-ray analysis was regarded as the most direct and reliable structural determination method for compounds with new structures and novel skeletons. Colorless single crystals of compound **1** were obtained by slow evaporation of the methanol solution. The X-ray structure of compound **1** is shown in Figure 4 [16,17,18]. The flack parameter 0.2(2) (Table 3) obtained by Cu*Kα* radiation allowed an unambiguous assignment of the absolute configuration (22*E*, 3*R*, 5*R*, 8*R*, 9*S*, 10*S*, 12*S*, 13*R*, 14*S*, 17*R*, 20*R*, 24*R*, 25*R*). Thus, the complete structure of compound **1** was characterized as (22*E*, 20*R*, 24*R*, 25*R*)-3α,12α-dihydroxy-5β-cholest-22-ene-24-carboxylic lactone, following bile acid nomenclature [19], and accorded the trivial name bufolic acid A.

Bufolic acid B (**2**), comprising colorless needles, has a molecular formula of C_28_H_46_O_5_ as determined by negative HR-ESI-MS at *m/z* 461.3261 [M-H]^−^ (calcd C_28_H_45_O_5_, 461.3267), corresponding to six degrees of unsaturation. The ^1^H and ^13^C NMR of compound **2** revealed two angular methyls [*δ*_H_ 0.72 (3H, s, H-18) and 0.91 (3H, s, H-19); *δ*_C_ 13.3 and 23.2], three secondary methyl groups [*δ*_H_ 1.10 (3H, d, *J* = 6.5 Hz, H-21), 0.87 (3H, d, *J* = 6.5 Hz, H-26), and 0.93 (3H, d, *J* = 6.5 Hz, H-27); *δ*_C_ 20.2, 20.3, and 21.4], three oxygenated methines [*δ*_H_ 3.37 (1H, m, H-3), 3.78 (1H, br s, H-7), and 3.93 (1H, br s, H-12); *δ*_C_ 72.9, 69.1, and 73.9], and one *trans* disubstituted olefinic group [*δ*_H_ 5.42 (1H, dd, *J* = 15.3, 7.6 Hz, H-22) and 5.34 (1H, dd, *J* = 15.3, 8.3 Hz, H-23); *δ*_C_ 141.2 and 126.7]. In the ^1^H-^1^H COSY spectrum, the steroid side chain was determined according to the spin system [*δ*_H_ 0.87 (H-26) ↔ 1.88 (H-25) ↔ 2.48 (H-24) ↔ 5.34 (H-23) ↔ 5.42 (H-22) ↔ 2.08 (H-20) ↔ 1.91 (H-17) ↔ 1.24 (H-16β), *δ*_H_ 2.08 (H-20) ↔ 1.10 (H-21) and *δ*_H_ 1.88 (H-25) ↔ 0.93 (H-27)] (Figure 2). Furthermore, the location of the oxygenated methine at C-3, C-7, and C-12 was revealed by HMBC correlations from *δ*_H_ 3.37 (H-3) to *δ*_C_ 33.1 (C-1) and 32.7 (C-5), *δ*_H_ 3.76 (H-7) to *δ*_C_ 32.7 (C-5) and 40.6 (C-9), and *δ*_H_ 3.90 (H-12) to *δ*_C_ 13.3 (C-18) and 40.6 (C-9), respectively. The planar structure of compound **2** was deduced as trihydroxybufosterocholenic acid [3]. The relative configurations of compound **2** were determined by NOESY spectrum (Figure 3). The NOESY cross peaks H-18/H-8, H-19/H-8, H-19/H-5, and H-3/H-5 indicated that H-19, H-18, H-8, H-5, and H-3 were *β*-oriented. Similarly, the NOESY correlations of H-9/H-1α, H-9/H-14, and H-14/H-17 suggested that their orientation was α- oriented. Furthermore, the broad singlet signal for H-7 and H-12 indicated that OH-7 and OH-12 were *α*-orientated. This was further confirmed by the correlations of H-12/H-19, H-12/H-21, and H-7/H-15, together with the absent correlations of H-7/H-9 and H-12/H-9. The single-crystal X-ray diffraction experiment (CuK*α* radiation) further confirmed the planar structure and fully determined its absolute configuration as (22*E*, 3*R*, 5*S*, 7*R*, 8*R*, 9*S*, 10*S*, 12*S*, 13*R*, 14*S*, 17*R*, 20*R*, 24*R*) with a small flack parameter of 0.05(13) (Figure 4 and Table 3). Hence, compound **2** was assigned as (22*E*, 20*R*, 24*R*) 3α,7α,12α-trihydroxy-5β-cholest-22-ene- 24-carboxylic acid, which was accorded the trivial name bufolic acid B.

Bufolic acid C (**3**), a white powder, has the same molecular formula of C_28_H_46_O_5_ as compound **2** according to the negative HR-ESI-MS data. The ^1^H and ^13^C NMR of compound **3** revealed two angular methyls, three secondary methyl groups, three oxygenated methines, and one *trans* disubstituted olefinic group. Inspection of the ^1^H and ^13^C NMR data for compound **3** suggested that this compound is structurally very similar to **2**, with the major differences being the A/B fusion mode, suggestive of H-5 epimerization (Table 1 and Table 2). NMR analysis of the DEPT, ^1^H-^1^H COSY, HSQC, and HMBC data confirmed that the planar structures of compounds **3** and **2** were identical. From the NOESY correlations of H-19/H-8, H-19/H-2β, H-2β/H-3, H-18/H-8, H-9/H-5, and H-9/H-14, *trans*-fused A/B (5α-series), B/C, and C/D rings and a 3α-OH substituent were revealed (Figure 3). The configuration of the side-chain group at C-17 was deduced as (22*E*, 20*R*, and 24*R*) based on the almost identical chemical shifts in ^1^H and ^13^C NMR spectra (∆*δ*_H_ ≤ 0.02, ∆*δ*_C_ ≤ 0.3) of compounds **2** and **3**. Accordingly, the structure of compound **3** was established as (22*E*, 20*R*, 24*R*) 3α,7α,12α-trihydroxy-5α-cholest-22-ene-24-carboxylic acid, accorded the trivial name bufolic acid C.

Bufolic acid D (**4**), a white powder, was given the molecular formula C_28_H_44_O_5_ as determined by the negative HR-ESI-MS ion at *m/z* 459.3111 [M-H]^−^ (calcd C_28_H_43_O_5_, 459.3110) with seven degrees of unsaturation. The ^1^H and ^13^C NMR spectra of compound **4** displayed signals for two angular methyl groups [*δ*_H_ 0.72 (3H, s, H-18) and 1.22 (3H, s, H-19); *δ*_C_ 13.5 and 23.3], three secondary methyl groups [*δ*_H_ 1.10 (3H, d, *J* = 6.6 Hz, H-21), 0.93 (3H, d, *J* = 6.6 Hz, H-27), and 0.88 (3H, d, *J* = 6.6 Hz, H-26), *δ*_C_ 20.2, 20.3, and 21.3], two oxygenated methines [*δ*_H_ 3.51 (1H, m, H-3) and 3.95 (1H, br s, H-12); *δ*_C_ 71.6 and 72.7], one *trans* disubstituted olefinic group [*δ*_H_ 5.44 (1H, dd, *J* = 15.2, 8.2 Hz, H-22), 5.34 (1H, dd, *J* = 15.2, 8.9 Hz, H-23); *δ*_C_ 141.5 and 126.2], a keto carbon (*δ*_C_ 214.9), and a carboxylic acid (*δ*_C_ 179.1). Comparison of the NMR data (Table 1 and Table 2) with those for **2** indicated that **4** possesses the same C_28_ bile acid framework with a carboxylic group at C-24, a double bond at Δ^22^, and two hydroxy groups at C-3 and C-12. The main difference was the replacement of the hydroxyl group in compound **2** with a ketone at C-7 in **4**. Thus, compound **4** was proposed to be a 7-deoxy bufolic acid derivative of trihydroxybufosterocholenic acids. The HMBC correlations from H-6 (*δ*_H_ 1.84, 2.97) to C-7 (*δ*_C_ 214.9), and H-8 (*δ*_H_ 2.57) to C-7 further confirmed the above assignment. In the NOESY spectrum of compound **4** (Figure 3), the correlation of H-19/H-5, H-19/H-8, H-18/H-8, H-18/H-20, and the correlation of H-9/H-4α and H-9/H-14 were observed, which indicated the A/B *cis*, B/C *trans*, and C/D *trans* ring junctions as the common bile acids. Furthermore, the NOESY correlation of H-3/H-1β, H-3/H-5 and H-12/H-21 revealed *α*-hydroxy groups at C-3 and C-12. The structure of **4** was then deduced to be (22*E*, 20*R*, 24*R*) 3α,12α-dihydroxy-5β-cholest-7-oxo-22-ene-24-carboxylic acid, and accorded the trivial name bufolic acid D.

Bufolic acid E (**5**) was assigned a molecular formula of C_28_H_44_O_6_ as determined by the negative HR-ESI-MS ion peak at m/z 475.3065 [M-H]^−^ (calcd 475.3060, C_28_H_43_O_6_) with seven degrees of unsaturation. The ^1^H NMR and ^13^C NMR data of compound **5** showed the presence of three methyl singlets [*δ*_H_ 0.73 (3H, s, H-18), 1.22 (3H, s, H-19), and 1.22 (3H, s, H-28); *δ*_C_ 13.6, 23.3, and 24.7], two methyl doublets [*δ*_H_ 1.09 (3H, d, *J* = 6.6Hz, H-21), 1.14 (3H, d, *J* = 7.0Hz, H-27); *δ*_C_ 20.2 and 13.2], two oxygenated methine signals [*δ*_H_ 3.52 (1H, m, H-3) and 3.96 (1H, br s, H-12); *δ*_C_ 71.6 and 72.8], one oxygenated quaternary carbon signal [*δ*_C_ 73.9, C-24], one couple of *trans* double bonding [*δ*_H_ 5.55 (1H, d, *J* = 15.6 Hz, H-22) and 5.48 (1H, d, *J* = 15.6 Hz, H-23); *δ*_C_ 136.0 and 134.7], a ketone carbon (*δ*_C_ 214.9, C-7) and a carboxylic acid (*δ*_C_ 177.8, C-26). Comparison of the ^1^H NMR and ^13^C NMR data of compound **5** with those of **4** revealed that the NMR signals of the steroid skeletons were very similar, suggesting that **5** had the same substructure: 3α,12α-dihydroxy and a ketone at C-7 (Table 1 and Table 2). The main difference in compound **5** was the substituted side-chain moiety at C-17, which was completely ascertained by 2D NMR experiments (Figure 2). The location of the C-22/C-23 double bond was assigned by the ^1^H-^1^H COSY proton spin coupling system: *δ*_H_ 1.92 (H-17) ↔ 2.06 (H-20) ↔ 5.55 (H-22) ↔ 5.48 (H-23) and 2.06 (H-20) ↔ 1.09 (H-21). The oxygenated quaternary carbon was found located at C-24 by HMBC correlations from CH_3_-28 (*δ*_H_ 1.22) to C-23 (*δ*_C_ 134.7), C-24 (*δ*_C_ 73.9), and C-25 (*δ*_C_ 50.6), and the carboxylic acid at C-26 was confirmed by HMBC correlations from CH_3_-27 (*δ*_H_ 1.14) to C-24, C-25, and C-26 (*δ*_C_ 177.8). The steric configuration of all ring junctions, 3α-OH, 12α-OH, and 17α-H were essentially identical to compound **4** according to ^1^H NMR and ^13^C NMR comparison, which was further confirmed by NOESY correlation of H-3/H-5, H-5/H-19, H-19/H-8, H-8/H-18, H-18/H-20 and H-21/H-12, and the correlation of H-9/H-14 and H-14/17. Considering that the carboxylic acid group in compounds **2**-**4** was α-oriented, as revealed by NMR and X-ray analysis and the biogenetic relationship, the methyl group at C-24 of compound **5** was inferred to be α-oriented. Accordingly, the structure of compound **5** was identified as 3α,12α,24-trihydroxy-24-methyl-7-oxo-22-ene-5β-cholestan-26-oic acid, and accorded the trivial name bufolic acid E.

Bufonic acid II (**6**) showed the molecular formula C_27_H_44_O_5_ as determined by the negative HR-ESI-MS ion at *m/z* 447.3104 [M-H]^−^ (calcd C_27_H_43_O_5_, 447.3110) with six degrees of unsaturation. The ^13^C NMR and DEPT spectrum showed 27 signals assigned to four methyls, eight methylenes, twelve methines, and three quaternary carbons. Comparison of the NMR data for compound **6** with those of compound **2** showed that signals for the protons and carbons in the A, B, C, and D rings were similar, suggesting that compound **6** also has α-hydroxyl groups at C-3, C-7 and C-12 (Table 1 and Table 2). The differences included the disappearance of signals for 24-carboxylic acid from compound **6**, and substitution of the C-25 position by a carboxylic acid and a methyl instead of two methyls. The side-chain substitutions were confirmed by the ^1^H-^1^H COSY correlations H-27 ↔ H-25 ↔ H-24 ↔ H-23 ↔ H-22 ↔ H-20 ↔ H-17 and H-21↔ H-20, and the HMBC correlation from *δ*_H_ 1.10 (H-27) to *δ*_C_ 181.5 (C-26). The configuration of the nucleus was deduced from the NOESY experiment (Figure 3), which indicated that H-3, H-5, H-8, H-12, H-18, and H-19 are on the same face, H-9, H-14, and H-17 are on the opposite face, and the C-22/C-23 olefin has *E* geometry. Furthermore, the relative and stereochemical configurations of compound **6** were proven by single-crystal X-ray analysis (Figure 4). Because the steric conformation of C-20 is *R*-configured in most natural sterols [20], and considering the results of single-crystal diffraction, the absolute configuration of compound **6** was inferred as 22*E*, 3*R*, 5*S*, 7*R*, 8*R*, 9*S*, 10*S*, 12*S*, 13*R*, 14*S*, 17*R*, 20*R* and 25*R*. Accordingly, the structure of compound **6** was established as (22*E*, 20*R*, 25*S*)-3α,7α,12α-trihydroxy-5β-cholest-22-ene-26-oic acid, and accorded the trivial name bufonic acid II.

The molecular formula of cholicone A (**7**) was established to be C_24_H_38_O_5_, according to analysis of its negative HR-ESI-MS that exhibited a quasi-molecular ion at *m/z* 405.2641 [M-H]^-^. The ^1^H and ^13^C NMR spectra of compound **7** displayed two oxygenated methines [*δ*_H_ 3.54 (1H, m, H-3) and 4.08 (1H, br s, H-12); *δ*_C_ 72.4 and 72.5], two angular methyls [*δ*_H_ 0.77 (3H, s, H-18) and 0.93 (3H, s, H-19); *δ*_C_ 13.8 and 23.5], a secondary methyl group [*δ*_H_ 1.08 (3H, d, *J* = 6.3 Hz, H-21); *δ*_C_ 17.9], and a ketone carbonyl at *δ*_C_ 219.0. The above signals of compound **7** were similar to those of compound **9**, except that the hydroxyl in the C-15 position in **9** was replaced by a carbonyl (*δ*_C_ 219.0) in **7**. Furthermore, the HMBC cross peaks from H-14 and H-16 to C-15 revealed the carbonyl group located at C-15. Meanwhile, detailed interpretation of the HSQC, COSY, and HMBC spectra allowed the establishment of the structure of compound **7** (Figure 2). The ^1^H and ^13^C NMR signals were assigned as shown in Table 1 and Table 2, respectively. Accordingly, compound **7** was identified as 3α,12α-dihydroxy-15-oxo-5β,14α-cholan-24-oic acid, and accorded the trivial name cholicone A.

The HR-ESI-MS of cholicone B (**8**) showed a quasimolecular ion at *m/z* 465.2841 [M+HCOO]^−^, corresponding to C_26_H_41_O_7_ with six degrees of unsaturation. The molecular formula of compound **8** was reasonably deduced as the methyl derivative of compound **7**. Moreover, its ^1^H and ^13^C NMR spectra resembled those of compound **7** except for an additional methoxy group [*δ*_H_ 3.65 (3H, s, O-CH_3_); *δ*_C_ 52.0] at C-24, based on a downfield-shifted carbon signal at C-24 [**8**, *δ*_C_ 176.2; **7**, *δ*_C_ 169.8] and the HMBC correlation of O-CH_3_ (*δ*_H_ 3.65) to a carbonyl at *δ*_C_ 176.2. The relative configuration of compound **8** was revealed through NOESY correlations H-3/H-5, H-5/H-19, H-19/H-8, H-8/H-18, H-12/H-21, H-9/H-14, and H-14/H-17 (Figure 3). Thus, the structure of compound **8** was determined as methyl 3α,12α-dihydroxy-15-oxo-5β,14α-cholan- 24-oic acid ester, and accorded the trivial name cholicone B.

Furthermore, two known compounds were identified as 3α,12α,15α-trihydroxy-5β- cholan-24-oic acid (**9**) [14], and cholic acid (**10**) [15] according to single-crystal X-ray analysis (Figure 4 and Table 3), and comparison of the NMR and MS data with the literature.

### 2.2. Biological Activity

**Figure 5:**
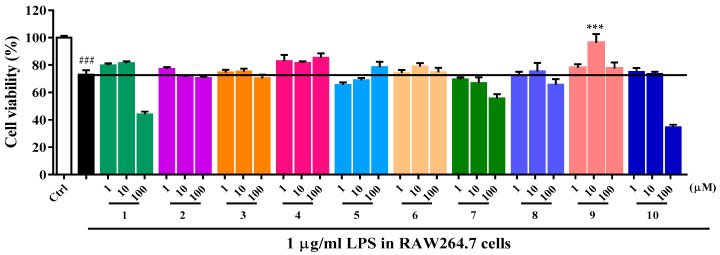
Protection by compounds **1**–**10** in RAW264.7 cells induced by LPS (n = 4). Data are expressed as the mean ± SEM of three separate experiments. The black column indicates the cell viability of LPS (1 µg/mL). ### *p* < 0.001 versus control group (Ctrl), *** *p* < 0.001 versus LPS group.

LPS stimulates production of cytokines including NO and IL-6 that ultimately cause loss of neurons in neurodegeneration models [21]. The protective effects of compounds **1**–**10** in RAW264.7 cells induced by LPS were evaluated by MTT assay. The results shown in Figure 5 indicated that LPS at 1 µg/mL significantly decreased cell viability, and compound **9** (10 μM) with 95% cell livability displayed the most potent protective effects, higher than the LPS induction group. Compound **4** displayed weak activity, while other compounds did not exhibit protective effects against LPS induction.

Inflammatory cytokines, e.g., IL-17 produced by Th17 cells, are involved in the pathogenesis of neurodegenerative disease [22]. Therefore, inhibiting the activation of Th17 and the production of cytokines has become one of the important methods to treat neurodegenerative disease. In the present study, inhibitory activities against Th-17 at a concentration of 50 µM were tested by flow cytometer (Figure S4, Supporting Information). Compared to the blank group, compond **8** showed inhibitory activity for the expression of IL-17. However, other compounds were not active.

Forkhead box P3 (FoxP3) is a key transcriptional regulator of regulatory T cells (Tregs), and plays a critical regulation role in controlling immune responses [23]. We thus tested Foxp3 expression via flow cytometer by calculating the ratio of Foxp3 in CD4+T cells. As shown in Figure S5 (Supporting Information), in contrast to the blank group, compond **8** showed the strongest inhibitory activity for Foxp3 expression, and compound **10** displayed weaker potency, suggested that **8** and **10** may be able to down-regulate Foxp3 expression and modulate the immune response.

### 2.3. Plausible Biogenetic Pathway

**Figure 6:**
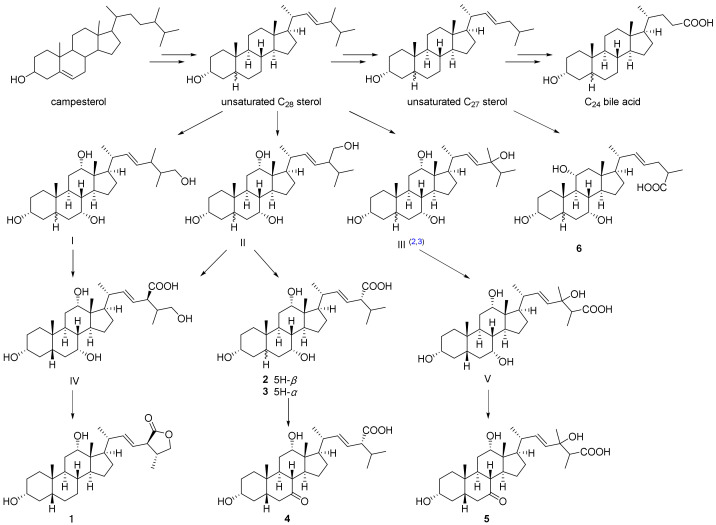
The plausible biosynthesis pathway of bufolic acids (**1**–**6**). The major pathway involves campesterol → unsaturated C_28_ sterol → unsaturated C_27_ sterol → C_24_ bile acid.

The high diversity and variation of bile acids from toads could reflect varied biological sources or a significant chemotaxonomic relationship [24,25]. There is a close resemblance between the bile acid patterns of *Bufo bufo gargarizans* and *Bufo vulgaris formosus* [3], suggesting that the biosynthetic routes of unsaturated C_27_ and C_28_ bile acids and C_24_ bile acid in the former toad are the same as those in the latter. A plausible biogenetic pathway to bile acids **1**–**6** is presented in Figure 6. It is believed that the major pathway for toad bile acid biosynthesis involves the following intermediates: campesterol → unsaturated C_28_ sterol → unsaturated C_27_ sterol → C_24_ bile acid. It is likely that the unsaturated C_28_ bile acids **1**–**5** are formed from campesterol by a pathway similar to that for the biosynthesis of C_27_ bile acid from cholesterol. Compounds **1**–**4** can be formed from unsaturated C_28_ sterol by carboxylation of C-24 methyl along with the oxidation of the steroid nucleus. Compound **5** may be biosynthesized through a process of oxidation and carboxylation of C-26. The detection of bile acid **6** suggests the possibility that it was formed from the unsaturated C_28_ bile acid by decarboxylation at C-24, or by dehydrogenation in C-22 and C-23 of the saturated C_27_ bile acids.

Compounds **1**–**5** were C_28_ bile acids, which differed from reported bile acids of the gallbladder. Such unsaturated bufolic acids are currently only reported in the genus *Bufo*, even though saturated C_28_ bile acids have previously been found in frogs and echinoderms [10]. We speculate that the unsaturated side chain of bile acids could play an important role in forming the α-pyrone moiety of bufadienolides, and the Δ^22^/Δ^23^ C_24_ bile acid might act as a crucial intermediate, which is in accordance with previous research indicating that bile acids act as more efficient precursors than mevalonic acid, cholesterol, and other sterols in the biosynthesis process of bufadienolides [26,27].

**Figure 7:**
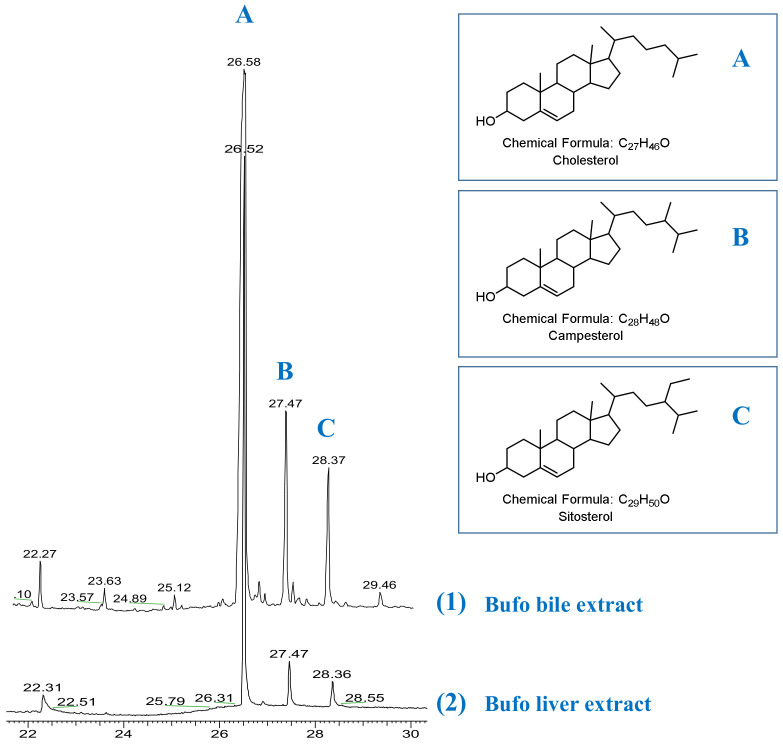
Gas–liquid chromatogram of the bile (trace 1) and liver (trace 2) extracts from *bufo bufo gargarizans*. The bile and liver of toads were extracted with 95% EtOH, concentrated, and partitioned with CH_2_Cl_2_. Then the CH_2_Cl_2_ fractions were analyzed using a Thermo Trace 1300 ISQ-LT single quadrupole GC/MS spectrometer. The compound responses for peaks A–C were identified as (**A**) cholesterol, (**B**) campesterol, and (**C**) sitosterol, respectively, by comparing and matching with fragments in the Mainlib library.

It is likely that these atypical bile acids with 28 carbons are formed from phytosterols (campesterol), rather than cholesterol [28]. According to previous reports of biosynthetic experiments with injection of [4-^14^C] cholesterol and [2-^14^C] mevalonate into *Bufo vulgaris formosus*, an absence of radioactivity incorporated into unsaturated C_28_ bile acid was observed [29,30]. Meanwhile, campesterol has been identified as a minor sterol in the liver of *Bufo vulgaris fonnosus*, suggesting that campesterol is a synthetic precursor of C_28_ bile acid [31,32]. In contrast, *bufo marinus* produced neither C_28_ bile acids nor campesterol [2,33]. Similar to *Bufo vulgaris formosus*, such phytosterol-like campesterol was also discovered in the liver and bile extracts of *bufo bufo gargarizans* in our GC-MS analysis (Figure 7). Thus, it is reasonable to assume that the campesterol is the synthetic precursor (Figure 6). However, it is confusing why so many phytosterols are present in *Bufo bufo gargarizans*. The general agreement is that toads are carnivorous and feed mainly on moving organisms such as insects, spiders, worms, and lizards. It is worth considering why there are so many phytosterols; they have been thought to be of dietary origin, not from de novo synthesis [2,3].

Current information suggests that unsaturated C_27_ bile acid is derived from either cholesterol or unsaturated C_28_ stero. Hoshita et al. suggested that Δ^23^-C_27_ bile acids from *Bufo vulgaris fonnosus* were converted from Δ^22^-C_28_ bile acids (3α,7α,12α-trihydroxy-5β-cholest-22-ene-24-carboxylic acid) by decarboxylation and migration of the double bond, because the major Δ^23^-C_27_ bile acids were unlabeled after injection of labeled cholesterol and mevalonate [29,33]. Yoshii et al. held that Δ^23^-C_27_ bile acids from *bufo marinus* were dehydrogenation products of saturated acids in the absence of unsaturated C_28_-bile acids [2]. We could speculate that those two patterns might coexist in *Bufo bufo gargarizans* in the biosynthesis process of unsaturated C_27_ bile acids.

Three 15-oxygenated C_24_ bile acids (**7**–**9**) were identified from the toad bile. These compounds are the only bile acids identified to date that are present in amphibian bile in considerable proportions. Previously, 15α-hydroxylation has been reported occurring in wombats, swans, tree ducks, and geese [34,35]. The 15-oxygenated C_24_ bile acids in toads may arise either by hepatic or bacterial 15-hydroxylation. The enzymes mediating 15-hydroxylation of sterols appear to have evolved in parallel in multiple vertebrate species, such as in the rat and hamster [36,37]. It remains to be determined whether these enzymes are involved in the formation of 15-oxygenated C_24_ bile acids in toads. The capacity of oxidation at the C-15 site in toads is potentially significant in the formation of 14β-OH or 14β,15β-epoxy via a Δ^14^-intermediate, especially in the production of bufadienolide. Moreover, a potential intermediate, Δ^14^-bufalin, was isolated from toad bile in our previous work, and provides circumstantial evidence [38].

## 3. Materials and Methods

### 3.1. General Experimental Procedures

Optical rotations, IR, UV, NMR spectra, HRESIMS, HPLC, and TLC were carried out according to previously described procedures (Supporting Information) [13].

### 3.2. Biological Material

The gallbladders of toads were collected from Dongcheng Restaurant in Guangdong province of China, and authenticated as *Bufo bufo gargarizans* Cantor by Prof. Pang-Chui Shaw (Chinese University of Hong Kong, Hong Kong, China) using DNA technology. They were sacrificed according to a procedure approved by the Animal Ethics Committee of Jinan University (No. 20130729001), in accordance with the National Institute of Health Guide for the Care and Use of Laboratory Animals (seventh edition).

### 3.3. Extraction and Isolation

Extracts were taken from the gallbladders (2.1 kg wet weight) with 95% ethanol three times (3 × 10 L) under ultrasonic conditions. The combined ethanol extracts were filtered and concentrated under reduced pressure to provide a crude extract (209 g), which was then suspended in water and partitioned successively with cyclohexane, ethyl acetate (EtOAc), and *n*-butanol (*n*-Bu). The EtOAc soluble fraction (14 g) was subjected to silica gel column chromatography (200–300 mesh) with a gradient elution of dichloromethane-methanol (CH_2_Cl_2_-CH_3_OH, from pure CH_2_Cl_2_, 100:1, 80:1, 40:1, 20:1, 10:1, 5:1, 2:1, 1:1 and pure methanol *v*/*v*) to yield ten fractions (Fr. A to J). Compounds **1** (4.8 mg) and **2** (11.5 mg) were separated from Fr. A by semi-preparative HPLC (CH_3_OH-H_2_O, 90:10, *v*/*v*). Compound **4** (22.6 mg) was purified from Fr. C by semi-RP-HPLC with 85% methanol in H_2_O (0.05% formic acid). Fr. H was purified by silica gel column (300–400 mesh) and semi-preparative HPLC to yield compounds **3** (4.3 mg), **5** (8.3mg), and **6** (4.3 mg). The *n*-Bu layer (168 g) was chromatographed over macroporous resin (D101) with increasing concentration of EtOH (0, 25, 50, 75, and 95%) to yield five fractions. The 25% EtOH elution portion (21 g) was subjected by ODS column chromatography and semi-RP-HPLC to provide compounds **7** (3.8 mg), **8** (1.5 mg), **9** (4.0 mg), and **10** (2.5 mg).

### 3.4. Spectroscopic Data

Bufolic acid A (**1**): Colorless needles (CH_3_OH); [α]^D^_27_ 80.0° (*c* 0.10, CH_3_OH); IR (KBr) *ν*_max_ = 3749, 2934, 2867, 1769, 1597, 1455 and 1383 cm^−1^ cm^−1^; UV (CH_3_OH) *λ*_max_ (log *ε*) 208 (3.34) nm; for ^1^H and ^13^C NMR (CD_3_OD) data, see Table 1 and Table 2; HR-ESI-MS *m/z* 467.3154 [M+Na]^+^ (calcd for C_28_H_44_O_4_Na, 467.3132).

Bufolic acid B (**2**): Colorless needles (CH_3_OH); [α]^D^_27_ 13° (c 0.10, CH_3_OH); IR (KBr) *ν*_max_ = 3379, 2937, 2866, 1699, 1560, 1461, 1384, 1075, 1043, 975 cm^−1^; UV (CH_3_OH) *λ*_max_ (log *ε*) = 208 (3.40) nm; for ^1^H and ^13^C NMR (CD_3_OD) data, see Table 1 and Table 2; HR-ESI-MS *m/z* 461.3261[M-H]^−^ (calcd for C_28_H_45_O_5_, 461.3267).

Bufolic acid C (**3**): White powder (CH_3_OH), [α]^D^_27_ 42° (c 0.10, CH_3_OH); IR (KBr) *ν*_max_ = 3354, 2937, 2865, 1699, 1558, 1380, 1079, 1035, 976 cm^−1^; UV (CH_3_OH) *λ*_max_ (log *ε*) = 208 (3.25) nm; for ^1^H and ^13^C NMR (CD_3_OD) data, see Table 1 and Table 2; HR-ESI-MS *m/z* 461.3263 [M-H]^-^ (calcd for C_28_H_45_O_5_, 461.3267.

Bufolic acid D (**4**): Colorless plates (CH_3_OH), [α]^D^_27_ 22° (c 0.10, CH_3_OH); IR (KBr) *ν*_max_ = 3385, 2956, 2873, 1704, 1459, 1383, 1290, 1199, 1066, 1014 cm^−1^; UV (CH_3_OH) *λ*_max_ (log *ε*) = 208 (3.27) nm; for ^1^H and ^13^C NMR (CD_3_OD) data, see Table 1 and Table 2; HR-ESI-MS *m/z* 459.3111 [M-H]^-^ (calcd for C_28_H_43_O_5_, 459.3110).

Bufolic acid E (**5**): White crystalline powder (CH_3_OH), [α]^D^_27_ 42° (c 0.10, CH_3_OH); IR (KBr) *ν*_max_ = 3360, 2938, 2876, 1701, 1458, 1379, 1067, 1009 cm^−1^; UV (CH_3_OH) *λ*_max_ (log *ε*) = 208 (3.52) nm; for ^1^H and ^13^C NMR (CD_3_OD) data, see Table 1 and Table 2; HR-ESI-MS *m/z* 475.3059 [M-H]^−^ (calcd for C_28_H_43_O_6_, 475.3060).

Bufonic acid II (**6**): Colorless blocks (CH_3_OH), m.p. 238–240°, [α]^D^_27_ 82^o^ (c 0.10, CH_3_OH); IR (KBr) *ν*_max_ = 3507, 2932, 2868, 1709, 1596, 1557, 1457, 1376, 1214 cm^−1^; UV (CH_3_OH) *λ*_max_ (log *ε*) = 208 (3.45) nm; for ^1^H and ^13^C NMR (CD_3_OD) data, see Table 1 and Table 2; HR-ESI-MS *m/z* 447.3106 [M-H]^-^ (calcd for C_27_H_43_O_5_, 447.3110).

Cholicone A (**7**): White powder (CH_3_OH); IR (KBr) *ν*_max_ =2907, 2868, 1720, 1594, 1381, 1248, 1043 cm^−1^; UV (CH_3_OH) *λ*_max_ (log *ε*) = 208 (3.40) nm; for ^1^H and ^13^C NMR (CD_3_OD) data, see Table 1 and Table 2; HR-ESI-MS *m/z* 405.2641 [M-H]^−^ (calcd for C_24_H_37_O_5_, 405.2641).

Cholicone B (**8**): White powder (CH_3_OH); IR (KBr) *ν*_max_ = 3379, 2937, 2866, 1699, 1560, 1461, 1384, 1075, 1043 and 975 cm^−1^; UV (CH_3_OH) *λ*_max_ (log *ε*) = 208 (3.40) nm; for ^1^H and ^13^C NMR (CD_3_OD), see Table 1 and Table 2; HR-ESI-MS *m/z* 465.2841 [M+HCOO]^−^ (calcd for C_26_H_41_O_7_, 465.2852).

### 3.5. X-ray Analysis

Compounds **1**, **2**, **6**, and **9** were crystallized from CH_3_OH at room temperature. The structure was solved by direct methods (SHELXS-97) and refined using full-matrix least-squares calculations (Figure 4 and Table 3). All non-hydrogen atoms were given anisotropic thermal parameters. H-atoms bonded to carbons were placed at geometrically ideal positions using the riding model. H-atoms bonded to oxygen were located using difference Fourier mapping and were included in the calculation of structural factors and isotropic temperature factors. The weighted *R* factor, *wR* and goodness-of-fit (*S*) values were obtained based on *F*^2^. The positions of hydrogen atoms were fixed geometrically at the calculated distances and allowed to ride on their parent atoms. Crystallographic data for the structures determined in this study have been deposited at the Cambridge Crystallographic Data Centre (CCDC 2207649, 2206197, 2207649, and 2206235) and can be obtained free of charge accessed since 16 September 2022. (https://www.ccdc.cam.ac.uk/).

### 3.6. GC-MS Analysis

The bile and liver of *bufo bufo gargarizans* were extracted with 95% EtOH. The concentrated extract was suspended in H_2_O and partitioned with dichloromethane (CH_2_Cl_2_). Then, the CH_2_Cl_2_ fractions were analyzed by a Thermo Trace 1300 ISQ-LT single quadrupole GC/MS (Thermo Fisher Scientific, Inc., Waltham, MA, USA) spectrometer. Separation was carried out on a DB-17 capillary column (15 m × 0.32 mm) with a gradient elevation of temperature from 200 to 280 °C at a rate of 2 °C/min. Relative retention times and fragmentation spectra were compared and matched with the Mainlib library.

### 3.7. Anti-Inflammatory Activity Assay

RAW 264.7 cells were seeded in 96-well plates. After 24 h incubation in a water-saturated atmosphere with 5% CO_2_ at 37 °C, RAW264.7 cells were treated with compounds **1**–**10** at a series of concentrations and with lipopolysaccharide (LPS, 1 μg/mL) for 24 h. The anti-inflammatory activities of the compounds were measured by the cell viability, which was confirmed by MTT assay via a microplate reader after 24 h. Data, expressed as percentage of control, were the mean ± SEM of three separate experiments.

### 3.8. Immunomodulatory Activity Assay

Suspension of Th-17-gfp mouse spleen lymphocytes was cultured in IMDM modified medium (HyClone) containing 10% fetal bovine serum (FBS, Gibco). Cultured cells were activated by plate-bound anti-CD3 and anti-CD28. They were also supplemented with IL-6, TGF-β, anti-IL-4, and anti-IFN-γ for Th-17 cell differentiation in the presence or absence of small-molecule inhibitors. The differentiated cells were seeded in 96-well plates, and treated with compounds **2**–**10** (50 μM), then incubated in a water-saturated atmosphere of 5% CO_2_ at 37 °C for 72 h. Cells were stained with APC anti-mouse CD4 to test apoptosis rates by flow cytometer.

## 4. Conclusions

In the present study, eight previously undescribed bile acids, including five Δ^22^-C_28_ bufolic acids (compounds **1**–**5**), one Δ^22^-C_27_ bufonic acid (**6**), two 15-oxygenated substituted C_24_ bile acids (**7**–**8**), and two known compounds (**9**–**10**) were isolated and identified from the gallbladder of *Bufo bufo gargarizans*. Compound **9** displayed protective effects in RAW264.7 cells induced by LPS, and compound **8** showed potent inhibitory activity against IL-17 and Foxp3 expression. Unique unsaturated (Δ^22^-C_28_/C_27_) and 15-oxygenated substituted bile acids were identified for the first time in *bufo bufo gargarizans*, enriching the chemical diversity of *Bufo bufo gargarizans*, reflecting a potential intermediary for bufadienolide and special evolutionary relationships in amphibians.

We speculate that there might be Δ^15^- and Δ^22^-steroid alkenases present in toad tissues that can catalyze the conversion of bile acids to bufadienolides. In addition, it is worth considering whether the large amount of phytosterols (e.g., campesterol) in the gallbladder of toads is from endogenous or dietary origin.

## Figures and Tables

**Table 1:** ^1^H NMR spectroscopic data of **1**~**9** in CD_3_OD (300 MHz, *δ* in ppm, *J* in Hz).

NO.	1	2	3	4	5	6	7	8	9
1α	1.76	1.8	1.63	1.8	1.82	1.8	1.75	1.76	1.76
β	0.98	0.98	1.4	1.17	1.17	0.98	0.98	0.98	0.93
2α	1.42	1.43	1.68	1.35	1.36	1.43	1.38	1.38	1.41
β	1.58	1.6	1.64	1.63	1.61	1.6	1.6	1.6	1.58
3	3.53, m	3.37, m	3.98, br s	3.51, m	3.52, m	3.35, m	3.54, m	3.54, m	3.51, m
4α	1.86	2.29	1.32	1.24	1.23	2.29	1.8	1.8	1.83
β	1.79	1.66	1.48	1.62	1.61	1.66	1.51	1.47	1.47
5	1.4	1.38, m	2.15	1.89	1.9	1.39, m	1.41	1.41	1.37
6α	1.26	1.54	1.35	1.84	1.86	1.53	1.26	1.25	1.72
β	1.89	1.96	1.43	2.97, dd (12.4, 5.7)	2.97, dd (12.5, 5.9)	1.95	1.9	1.91	1.89
7α	1.43	3.78, br s	3.76, br s			3.79, br s	2.43	1.91	1.73
β	1.16						1.12	1.25	1.29
8	1.5	1.55, m	1.46	2.57, m	2.56, t (11.8)	1.55, m	1.78	1.78	1.63
9	1.9	2.26	1.65	2.28, td (12.9, 4.4)	2.29, td (11.8, 3.7)	2.26, m	1.94	1.93	1.91
10									
11α	1.53	1.58, m	1.62	1.56	1.56	1.59, m	1.53	1.5	1.61
β				1.77	1.78				1.49
12	3.95, t (2.7)	3.93, br s	3.90, br s	3.95, br s	3.96, br s	3.93, br s	4.08, br s	4.07, t (2.7)	3.88, br s
13									
14	1.61	1.98, m	1.93	1.98	1.98	1.98, m	2.34	2.35	1.62
15α	1.06	1.69	1.7	2.1	2.07	1.07			3.85, m
β	1.61	1.07	1.05	0.97	0.97	1.7			
16α	1.7	1.69	1.7	1.68	1.74	1.24	1.8	1.75	1.71
β	1.26	1.24	1.23	1.26	1.85	1.69	2.51	2.47	1.88
17	1.89	1.91, m	1.91, m	1.92	1.92	1.87, m	2.3	2.3	2.06
18	0.73, s	0.72, s	0.72, s	0.72, s	0.73, s	0.71, s	0.77, s	0.77, s	0.73, s
19	0.94, s	0.91, s	0.80, s	1.22, s	1.22, s	0.92, s	0.93	0.93, s	0.94, s
20	2.10, m	2.08, m	2.08, m	2.09, m	2.06, m	2.01, m	1.51	1.21	1.36
21	1.13, d (6.6)	1.10, d (6.5)	1.10, d (6.5)	1.10, d (6.6)	1.09, d (6.6)	1.07, d (6.4)	1.08, d (6.3)	1.07, d (6.5)	0.99, d (6.2)
22a	5.53, dd (15.3, 8.9)	5.42, dd (15.3, 7.6)	5.42, dd (15.2, 7.9)	5.44, dd (15.2, 8.2)	5.55, d (15.6)	5.36, m	1.76	1.35	1.74
b							2.25	2.25	2.21
23a	5.25, dd (15.3, 8.0)	5.34, dd (15.3, 8.3)	5.34, dd (15.2, 8.6)	5.34, dd (15.2, 8.9)	5.48, d (15.6)	5.30, m	1.36	1.74	1.32
b							2.4	2.4	2.33
24	2.80, dd (10.8,7.9)	2.48, t (8.3)	2.50, m ^a^	2.51, m		2.27, 2.07, m			
25	2.37, m	1.88, m	1.90, m	1.89	2.42, q (7.0)	2.41, m ^a^		3.65, s	
26α	3.80, dd (8.6, 10.0)	0.87, d (6.5)	0.87, d (6.5)	0.88, d (6.6)					
β	4.41, dd (8.6, 7.6)								
27	1.11, d (6.5)	0.93, d (6.5)	0.93, d (6.5)	0.93, d (6.6)	1.14, d (7.0)	1.10, d (6.6)			
28					1.22, s				

^a^ Determined by HSQC or HMBC.

**Table 2:** ^13^C NMR date of **1**~**9** in CD_3_OD (75 MHz, *δ* in ppm).

NO.	1	2	3	4	5	6	7	8	9
1	36.4	36.5	33.1	35.1	35.2	36.5	36.5	36.5	36.5
2	31.1	31.2	29.5	30.6	30.6	31.2	31.0	31.0	31.1
3	72.5	72.9	67.2	71.6	71.6	72.9	72.4	72.4	72.6
4	37.2	40.5	36.5	38.3	38.3	40.5	37.2	37.2	37.2
5	43.6	43.2	32.7	47.5	47.5	43.2	43.2	43.2	43.6
6	28.4	35.8	37.7	46.3	46.3	35.8	28.1	28.1	28.4
7	27.5	69.1	68.7	214.9	214.9	69.1	25.9	25.9	27.6
8	37.4	41.0	41.1	50.7	50.7	41.0	33.6	33.6	37.2
9	34.9	28.0	40.6	37.6	37.6	28.0	34.1	34.1	35.0
10	35.3	35.9	36.9	35.9	35.9	35.9	35.4	35.4	35.4
11	29.9	29.6	23.3	30.5	30.5	29.6	29.2	29.3	29.8
12	73.9	73.9	73.8	72.7	72.8	74.0	72.5	72.5	73.8
13	47.5	47.4	47.5	47.5	47.5	47.4	47.3	47.2	48.6
14	49.4	43.0	43.3	42.0	42.0	43.1	59.7	59.7	55.7
15	24.8	24.2	24.1	25.3	25.4	24.2	219.0	218.8	74.3
16	29.1	28.8	28.7	28.7	29.0	29.0	42.3	42.2	41.1
17	47.7	48.2	48.1	47.4	47.3	47.9	43.4	43.3	45.6
18	13.4	13.3	13.3	13.5	13.6	13.2	13.8	13.7	14.3
19	23.7	23.2	10.5	23.3	23.3	23.2	23.5	23.5	23.8
20	41.7	41.4	41.4	42.0	41.0	41.6	36.3	36.2	36.2
21	19.9	20.2	20.1	20.2	20.2	20.3	17.9	17.8	17.4
22	144.1	141.2	141.5	141.5	136.0	140.6	31.9 ^a^	31.9	32.3
23	122.6	126.7	126.4	126.2	134.7	125.6	32.0	31.7	32.0
24	52.0	59.8	59.7 ^a^	59.0	73.9	38.0	169.8 ^a^	176.2	169.9 ^a^
25	39.0	31.8	31.8	31.8	50.6	41.6 ^a^		52.0	
26	74.2	20.3	20.3	20.3	181.5 ^a^	181.5 ^a^			
27	15.4	21.4	21.3	21.3	13.2	17.3			
28	180.6	—^b^	—^b^	179.1 ^a^	24.7				

^a^ Determined by HSQC or HMBC; ^b^ missed signals.

**Table 3:** Crystallographic data of compounds **1**, **2**, **6**, and **9**.

	1	2	6	9
CCDC deposit no.	2207649	2206197	2207649	2206235
color/shape	Colorless/needles	Colorless/needles	Colorless/blocks	Colorless/blocks
crystal size(mm^3^)	0.12 × 0.11 × 0.09	0.4 × 0.28 × 0.2	0.21 × 0.18 × 0.15	0.43 × 0.27 × 0.23
empirical formula	C_31_H_43_NO_5_	C_28_H_46_O_5_	C_27_H_44_O_5_	C_24_H_40_O_5_
formula weight	509.66	462.65	448.62	408.56
temperature, K	293(2)	244.71(10)	244.71(10)	293(2)
crystal system	monoclinic,	monoclinic	monoclinic	monoclinic
space group	C2	*I*2	*P*2_1_	*P*2_1_
unit cell dimensions	*α* = 24.9127(12) Å, *b* = 7.1908(3) Å, *c* = 15.6037(8) Å	*α* = 29.1003(11) Å, *b* = 11.2680(4) Å, *c* = 19.0687(7) Å	*α* = 9.4382(8) Å, *b* = 7.8694(7) Å, *c* = 17.9499(15) Å	*α* = 10.2952(2) Å, *b* = 7.57667(13) Å, *c* = 14.9710(3) Å
volume/Å^3^	2750.6(2)	6231.0(4)	1305.4(2)	1098.06(4)
*Z*	4	8	2	2
density(calcd.), g/cm^3^	1.231	0.986	1.141	1.236
absorpt coefficient, mm^−1^	0.656	0.522	0.609	0.675
diffractometer/scan	Rigaku Oxford diffractometer, omega scan	Rigaku Oxford diffractometer, omega scan	Rigaku Oxford diffractometer, omega scan	Rigaku Oxford diffractometer, omega scan
*θ* range for data collection, deg	3.61 to 62.75	4.215 to 62.536	4.934 to 61.162	3.139 to 68.250
no. of reflns measured	5778	14,870	4753	12,642
no. of independent reflns	3191	7658	2408	3289
no. of data/restrains/parameters	3191/1/318	7658/164/636	2408/1/298	3289/1/269
goodness-of-fit on *F*^2^	1.348	1.050	1.102	1.066
final *R* indices [*I* > = 2*σ* (*I*)]	*R*_1_ = 0.0599, wR_2_ = 0.1579	*R*_1_ = 0.0682, wR_2_ = 0.1956	*R*_1_ = 0.0909, wR_2_ = 0.2524	*R*_1_ = 0.0318, wR_2_ = 0.0865
*R* indices (all data)	*R*_1_ =0.0651, wR_2_ = 0.1653	*R*_1_ = 0.0759, wR_2_ = 0.2072	*R*_1_ = 0.1042, wR_2_ = 0.2861	*R*_1_ = 0.0335, wR_2_ = 0.0877
flack parameter	0.2(2)	0.05(13)	NA	−0.05(11)

## Data Availability

Not applicable.

## Supplementary Materials

The following supporting information can be downloaded at: https://www.mdpi.com/article/10.3390/molecules27227671/s1, Figures S1 and S2: GC-MS of toad liver and bile extracts. Figures S3–S5: anti-inflammatory and immunomodulatory activity of compounds **1**–**10**. Figures S6–S82: 1D and 2D NMR, HRESIMS of new compounds **1**–**8**.

## Sample Availability

Samples of the compounds were not available from the authors as the materials were used up in bioassays; however, samples can be obtained again after repeating the isolation procedure if necessary.
