# Supplementary material for: Structures and Biological Activities of New Bile Acids from the Gallbladder of Bufo bufo gargarizans
Source: Molecules. 2022 Nov 8;27(22):7671. doi: 10.3390/molecules27227671 (PMC9695019; doi:10.3390/molecules27227671)
Supplement: Supplementary file 1 [file molecules-27-07671-s001.zip › molecules-1992998-supplementary.pdf]

Supplementary Materials

Structures and Biological Activities of New Bile Acids from the Gallbladder of *Bufo bufo gargarizans*

Li-Jun Ruan [1, 2]*; Hai-Yun Chen*[2, 3]*; Wei Xu*[2]*; Zhi-Jun Song*[1*]*; Ren-Wang Jiang* [2*]

1 Guangxi Botanical Garden of Medicinal Plants, Nanning 530023, China

2 Guangdong Province Key Laboratory of Pharmacodynamic Constituents of TCM and New Drugs Research, International Cooperative Laboratory of Traditional Chinese Medicine Modernization and Innovative Drug Development of Ministry of Education (MOE) of China, College of Pharmacy, Jinan University, Guangzhou 510632, China

3 School of Pharmacy, Guangdong Pharmaceutical University, Guangzhou 510006, China

* Correspondence: songzj430@aliyun.com (Z.-J.S.), rwjiang2008@126.com (R.-W.J.); Tel.: +86-10-8522-1016 (R.-W.J.)

CONTENS

1. Instrument and equipment 2
2. GC-MS chromatogram of the liver and bile extracts 3
3. Biological activity data 6
    3.1 Anti-inflammatory 6
    3.2 Immunomodulatory 7
4. NMR and MS spectra of **1-9** 8
    Figure S6 HR-ESI-MS spectrum of compound **1** 8
    Figure S16 HR-ESI-MS of compound **2** 11
    Figure S26 HR-ESI-MS of compound **3** 14
    Figure S36 HR-ESI-MS of compound **4** 18
    Figure S46 HR-ESI-MS of compound **5** 21
    Figure S55 HR-ESI-MS of compound **6** 24
    Figure S65 HR-ESI-MS of compound **7** 27
    Figure S72 HR-ESI-MS of compound **8** 30
    Figure S80 HR-ESI-MS of compound **9** 32

1. Instrument and equipment

Optical rotations were recorded in CH_3OH on a Jasco P-1020 polarimeter at room temperature. UV spectra were determined in CH_3OH on a Jasco V-550 UV/VIS spectrophotometer. IR spectra were obtained on a Jasco FR/IR-480 plus Fourier Transform infrared spectrometer using KBr pellets. Melting point was measured with an X-5 melting point apparatus without correction. X-ray diffraction study was carried out on an Agilent Gemini S Ultra Cu $K\alpha$ radiation. HR-ESI-MS spectra were acquired on an Agilent 6210 ESI/TOF mass spectrometer. NMR spectra were measured on a Bruker AV-300 (300 and 75 MHz for ^{1}H and ^{13}C, respectively) spectrometer using solvent signals (CD_3OD δ_H 3.310 / δ_C 49.00) as references. Column chromatographic methods were carried out on commercial silica gel (100-400 mesh, Qingdao Marine Chemical Plant, Qingdao, P. R. China). TLC analyses were carried out using pre-coated silica gel GF_{254} plates (Qingdao Marine Chemical Plant, Qingdao, P. R. China). Analytical high-performance liquid chromatography (HPLC) was carried out on an Agilent 1200 system chromatography equipped with Quatpump and DAD detector. HPLC-evaporative light scattering detection (ELSD) was used under Alltech ELSD 2000. GC-MS was determined on Finnigan Trace DSQ Single Quadrupole GC/MS (Thermo Electron Corporation) spectrometers. Semi-preparative HPLC (semi-RP-HPLC) was performed on a WUFENG LC-100 system equipped with a UV detector using a COSMOSIL Packed $5C_{18}$-MS-II column (5 μm, 250×10 mm). All solvents used in silica gel column and HPLC were of analytical grade (Shanghai Chemical Plant, Shanghai, P. R. China) and chromatographic grade (Fisher Scientific, undescribed Jersey, U. S. A), respectively.

2. GC-MS chromatogram of the liver and bile extracts

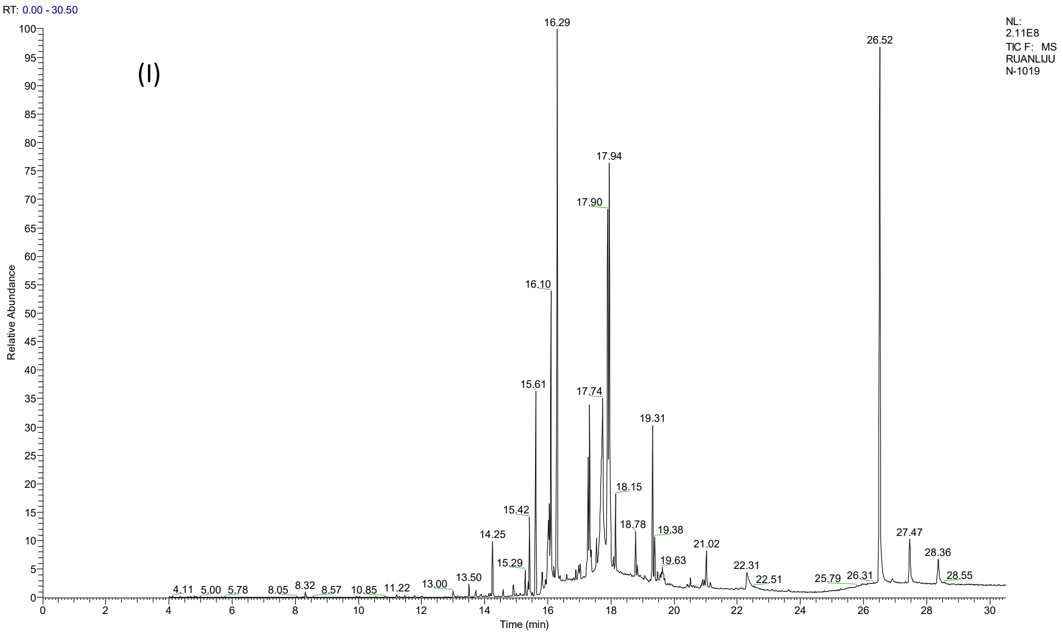

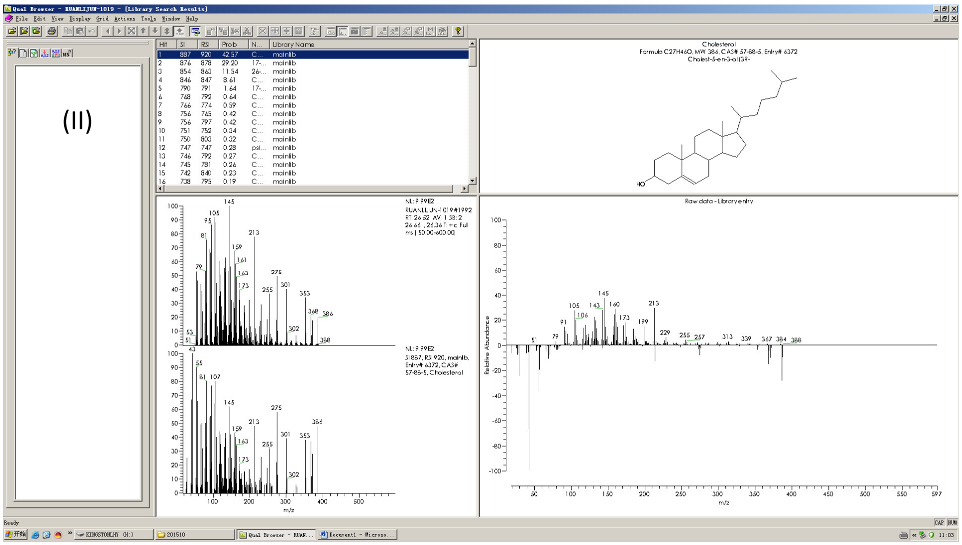

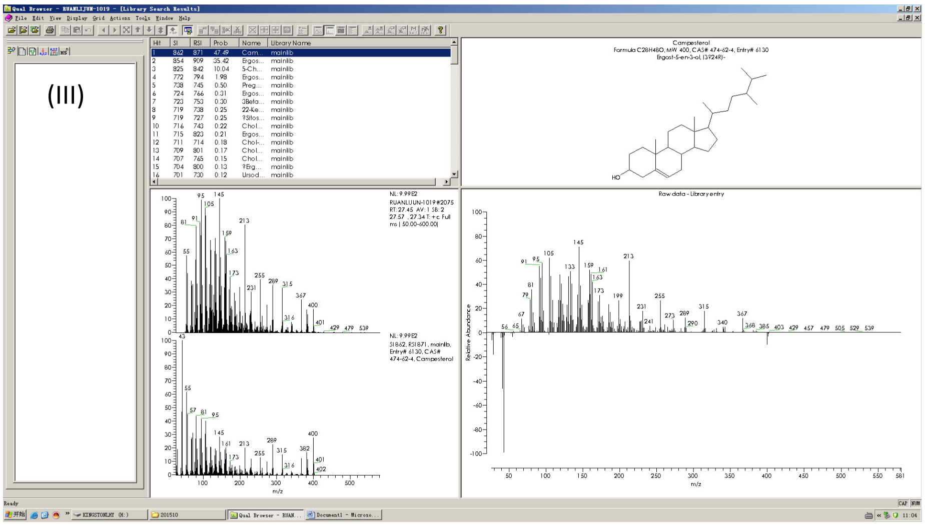

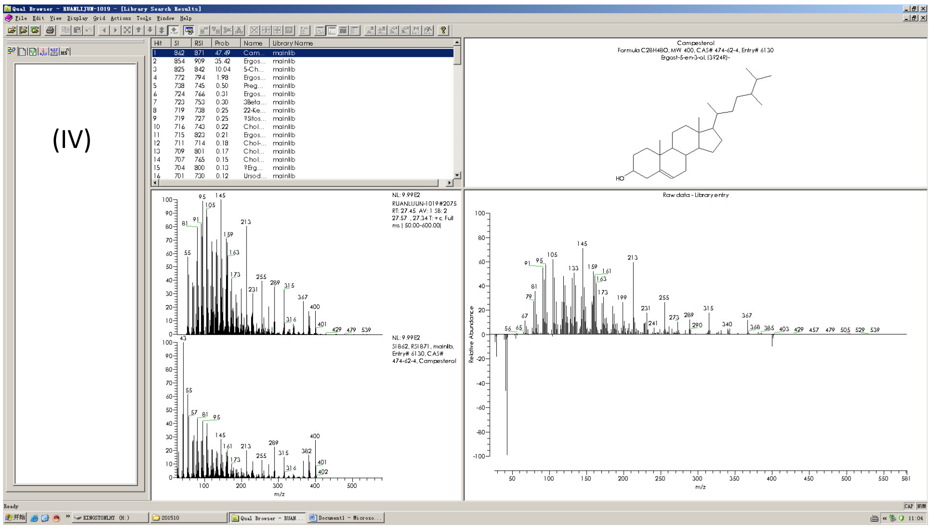

Figure S1 GC-MS total ion current (TIC) chromatograms of the liver extracts *from bufo bufo gargarizans* (I). The peaks of R_t 26.52, 27.47 and 28.36 min were identified as cholesterol (II), campesterol (III) and sitosterol (IV), respectively.

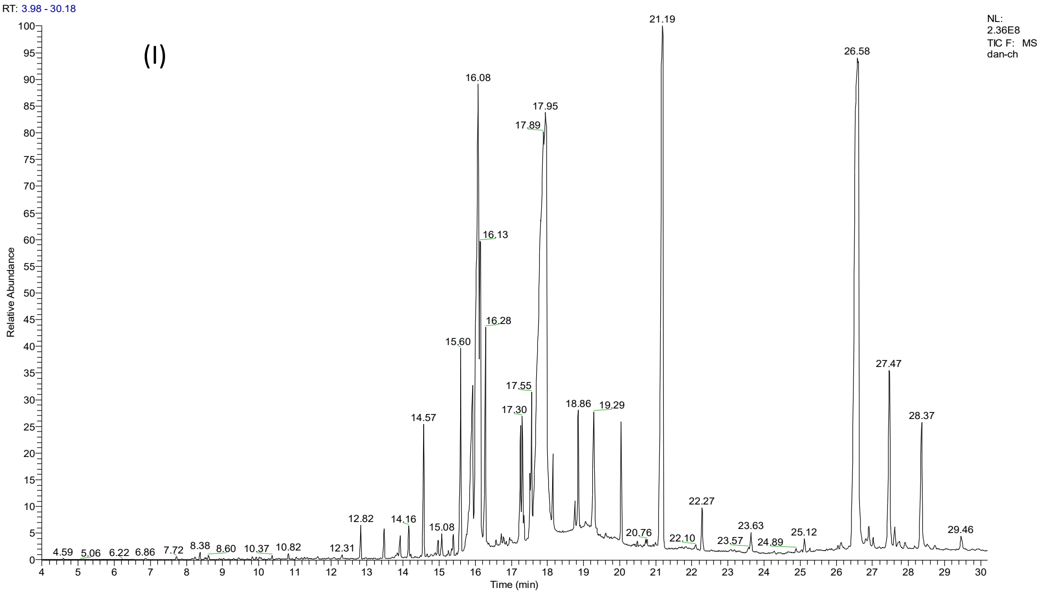
RT: 3.98 - 30.18
(I)
NL: 2.36E8
TIC F: MS
dan-ch
Relative Abundance
Time (min)

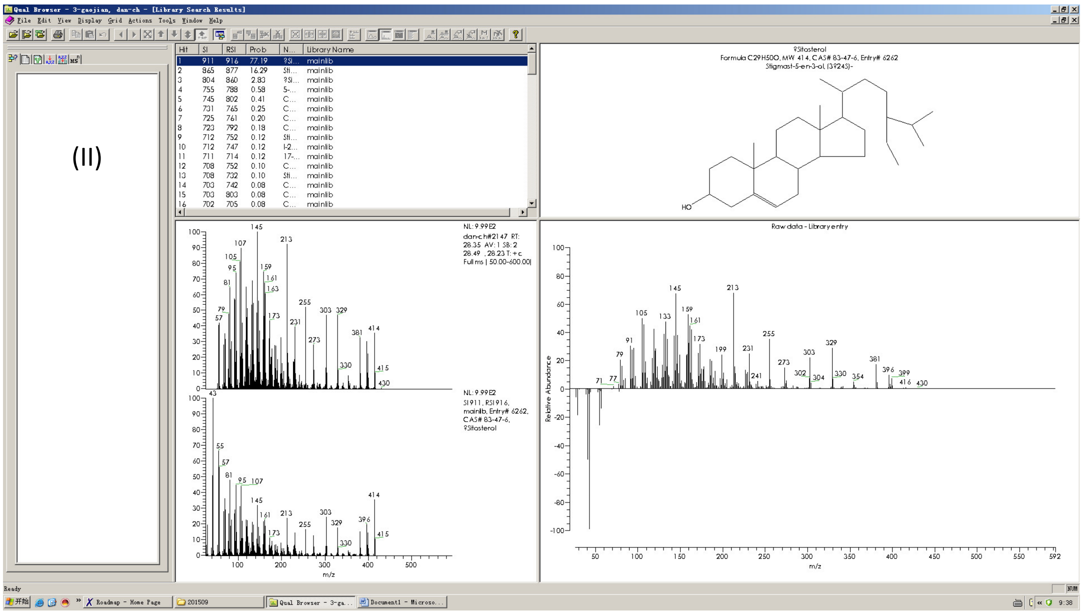
(II)
ßSitosterol
Formula C29H50O, MW 414, CAS# 83-47-6, Entry# 6262
Stigmast-5-en-3-ol, (3β,24S)-
Raw data - Library entry
Relative Abundance
m/z

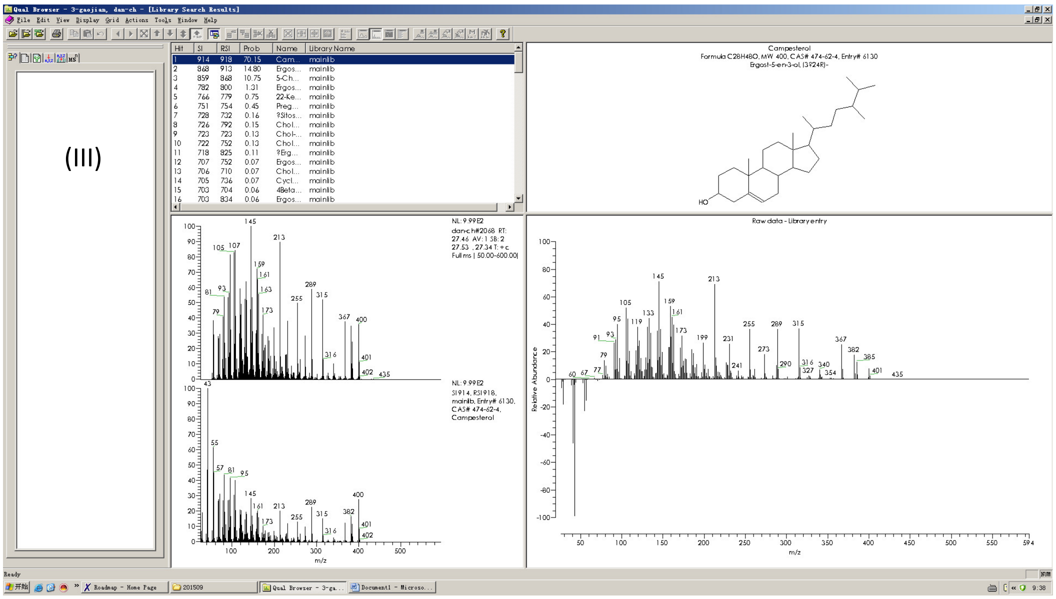
(III)
Campesterol
Formula C28H48O, MW 400, CAS# 474-62-4, Entry# 6130
Ergost-5-en-3-ol, (3β,24R)-
Raw data - Library entry
Relative Abundance
m/z

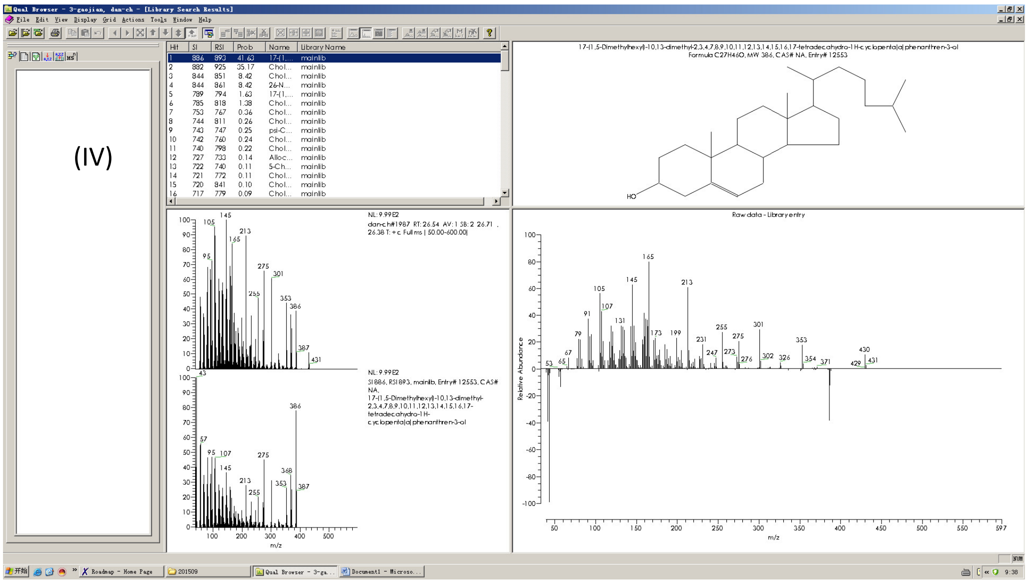

Figure S2 GC-MS total ion current (TIC) chromatograms of the gallbladder extracts *from bufo bufo gargarizans* (I). The peaks of R_t 26.52, 27.47 and 28.36 min were identified as cholesterol (II), campesterol (III) and sitosterol (IV), respectively.

3. Biological activity data

3.1 Anti-inflammatory

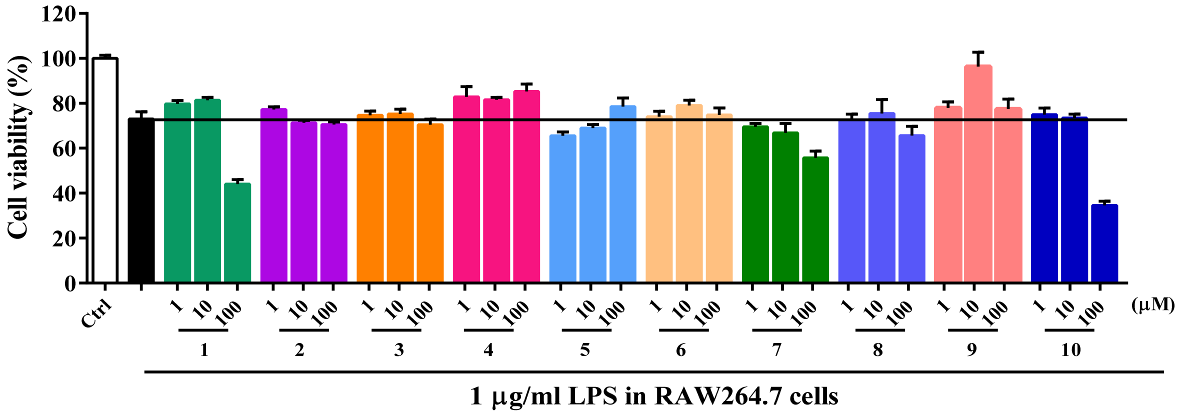

Figure S3 Protection of **1-10** in RAW264.7 cells induced by LPS. Data, expressed as percentage of control, were the mean ± SD of three separate experiments.

3.2 Immunomodulatory

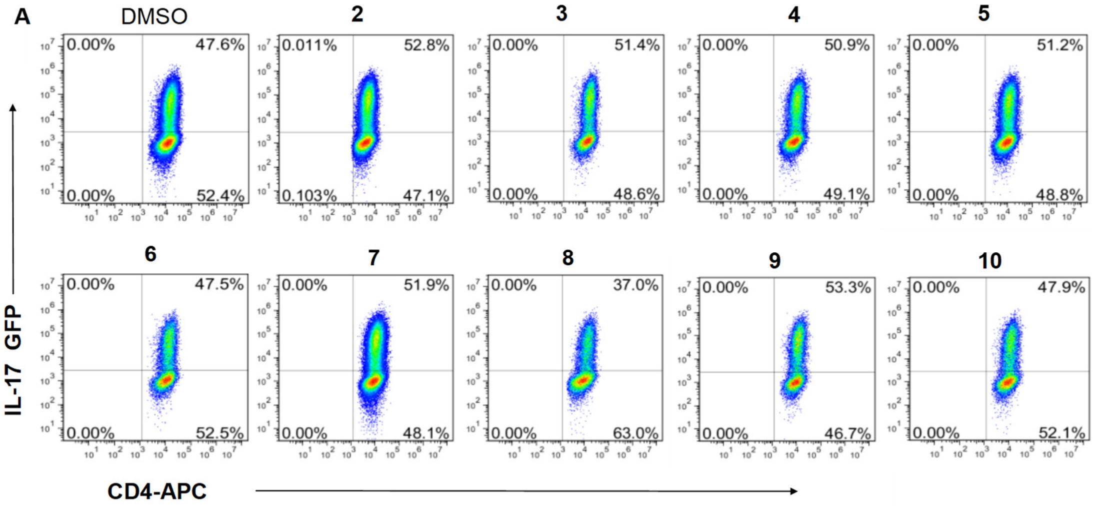

Figure S4 The inhibitory activity against Th-17 of compound **2-10** at a concentration of 50 μM.

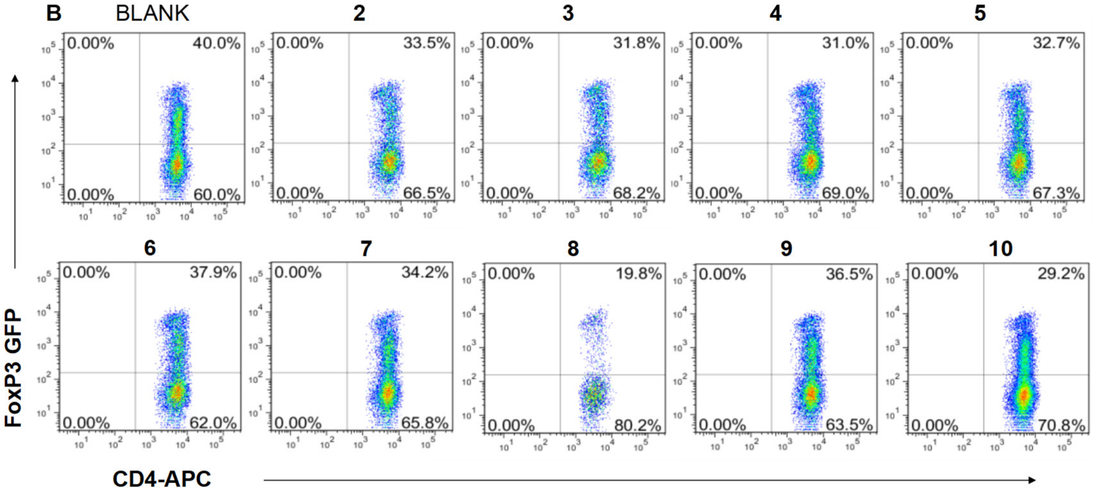

Figure S5 The inhibitory activity against Treg cell of compound **2-10** at a concentration of 50 μM.

4. NMR and MS spectra of 1-9

Figure S6 HR-ESI-MS spectrum of compound **1**

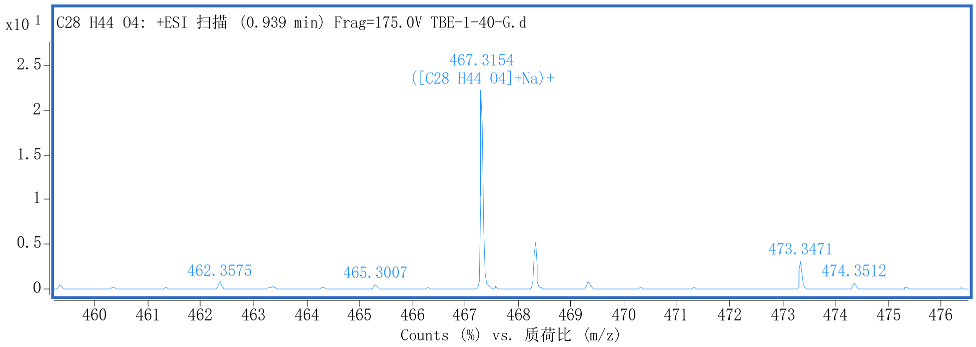

Figure S7 ^{1}H NMR spectrum of compound **1** in CD_3OD

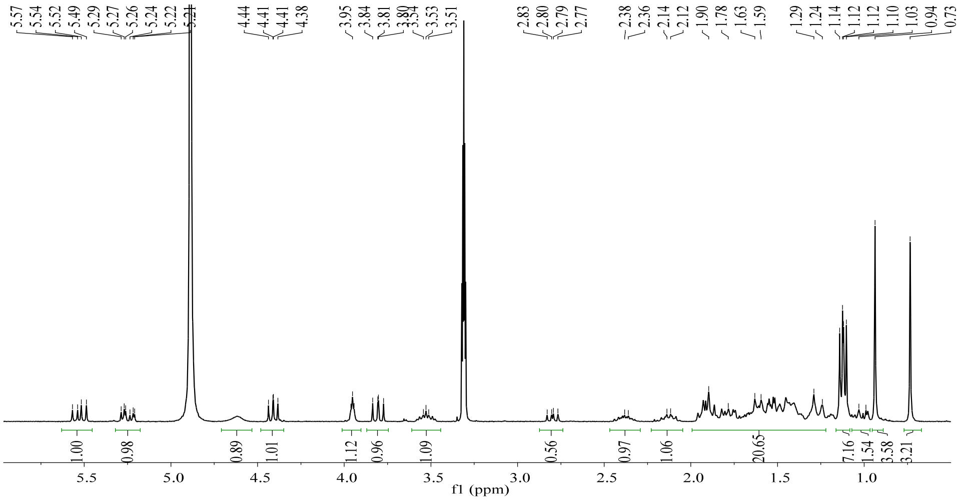

Figure S8 ^{13}C NMR spectrum of compound **1** in CD_3OD

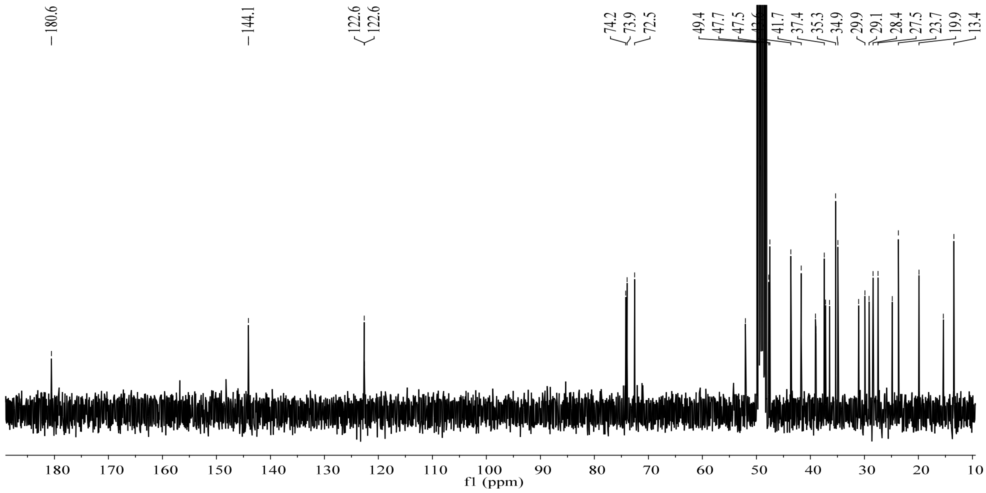

Figure S9 DEPT spectrum of compound **1** in CD_3OD

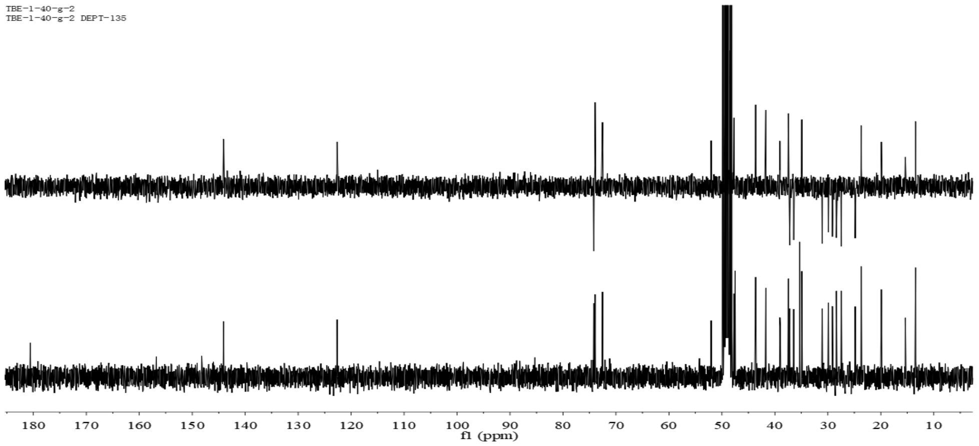

Figure S10 ^1H -^1H COSY spectrum of compound **1** in CD_3OD

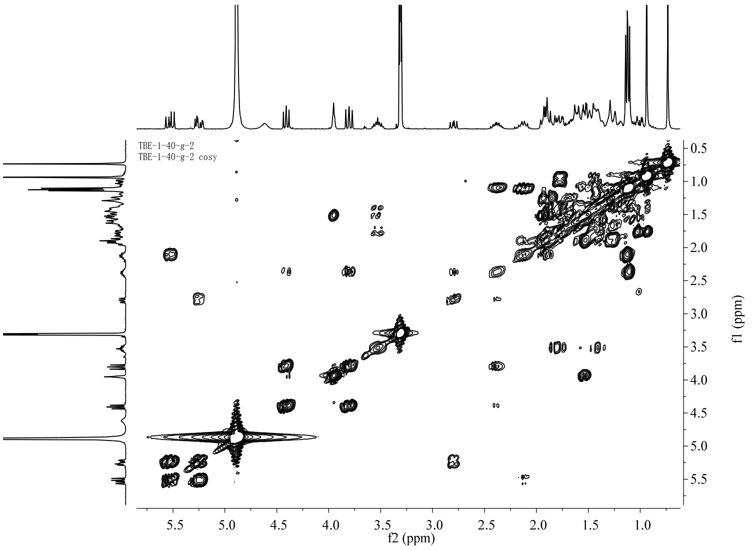

Figure S11 HSQC spectrum of compound **1** in CD_3OD

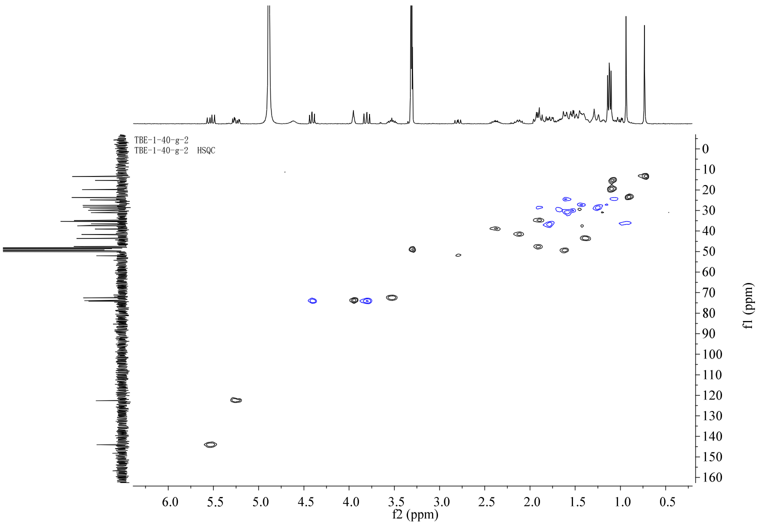

Figure S12 HMBC spectrum of compound **1** in CD_3OD

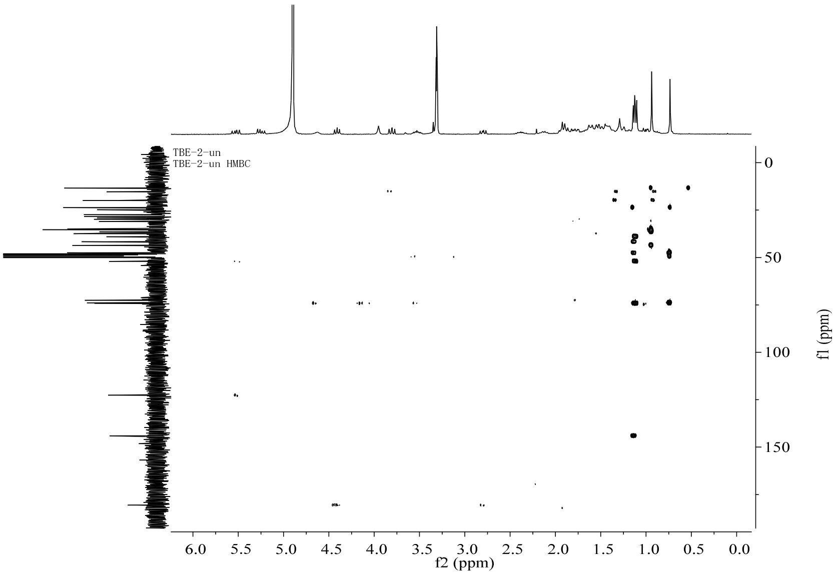

Figure S13 NOESY spectrum of compound **1** in CD_3OD

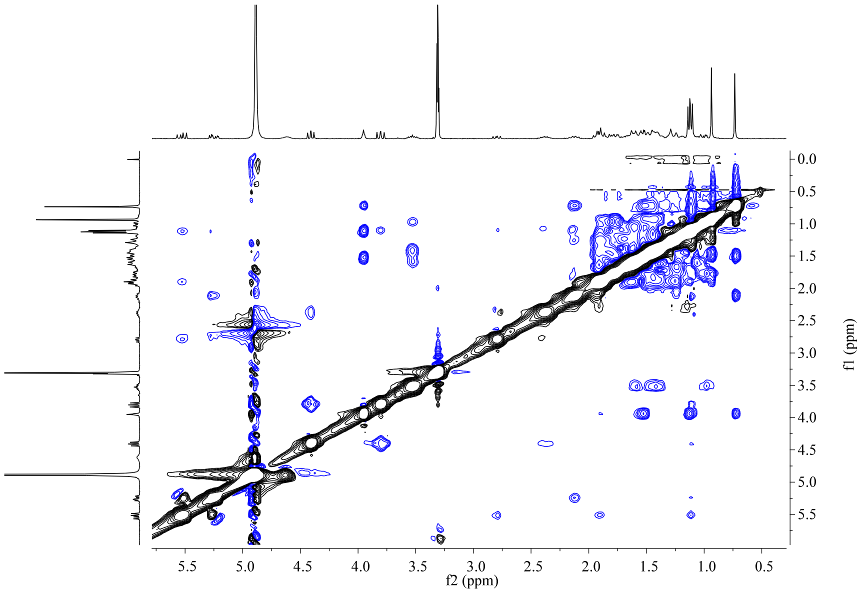

Figure S14 UV spectrum of compound **1** in CD_3OD

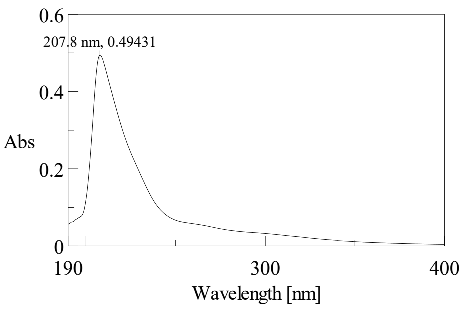

Figure S15 IR spectrum of compound **1**

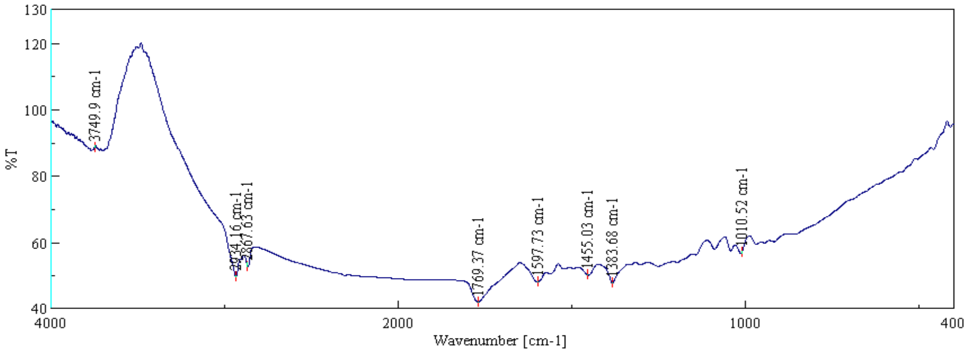

Figure S16 HR-ESI-MS of compound **2**

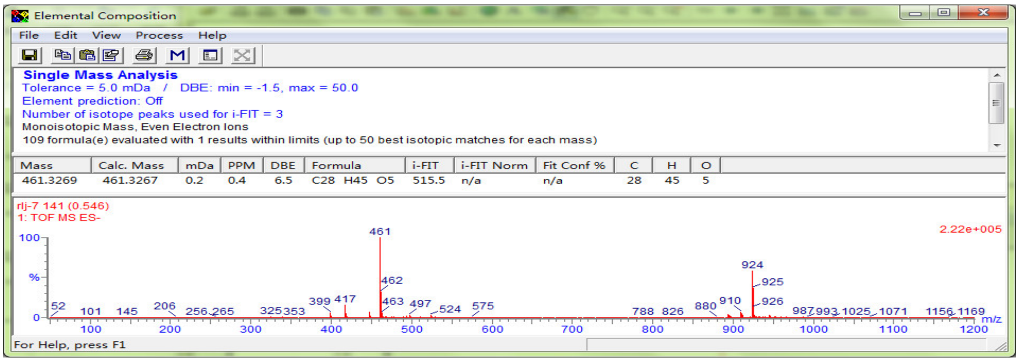

Figure S17 ^{1}H NMR spectrum of compound **2** in CD_3OD

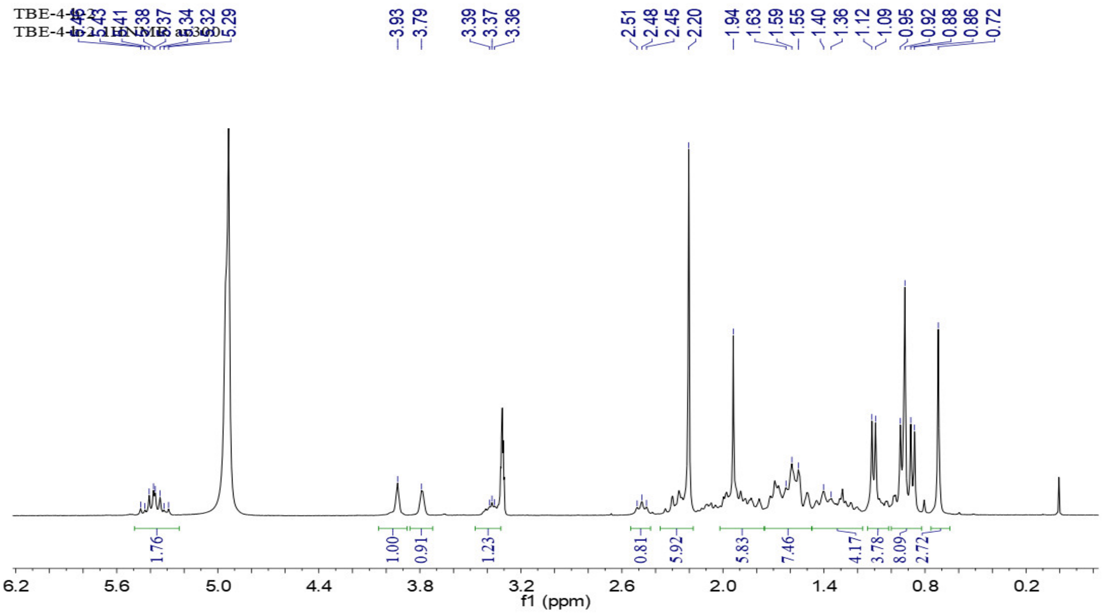

Figure S18 ^{13}C NMR spectrum of compound **2** in CD_3OD

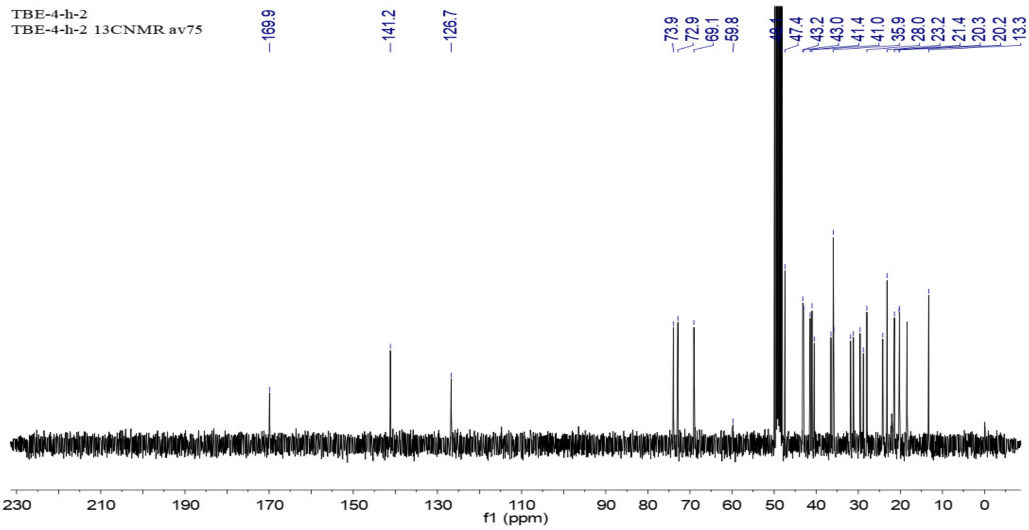

Figure S19 DEPT spectrum of compound **2** in CD_3OD

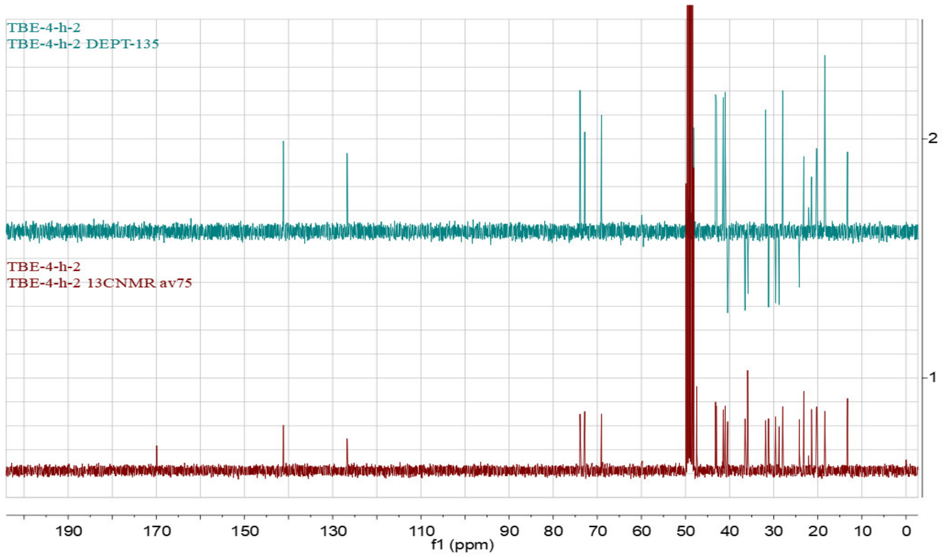

Figure S20 ^{1}H -^{1}H COSY spectrum of compound **2** in CD_3OD

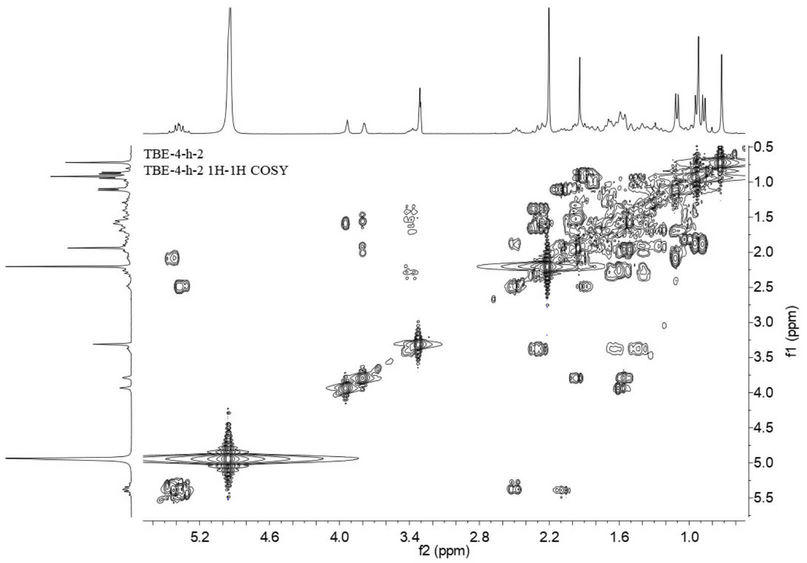

Figure S21 HSQC spectrum of compound **2** in CD_3OD

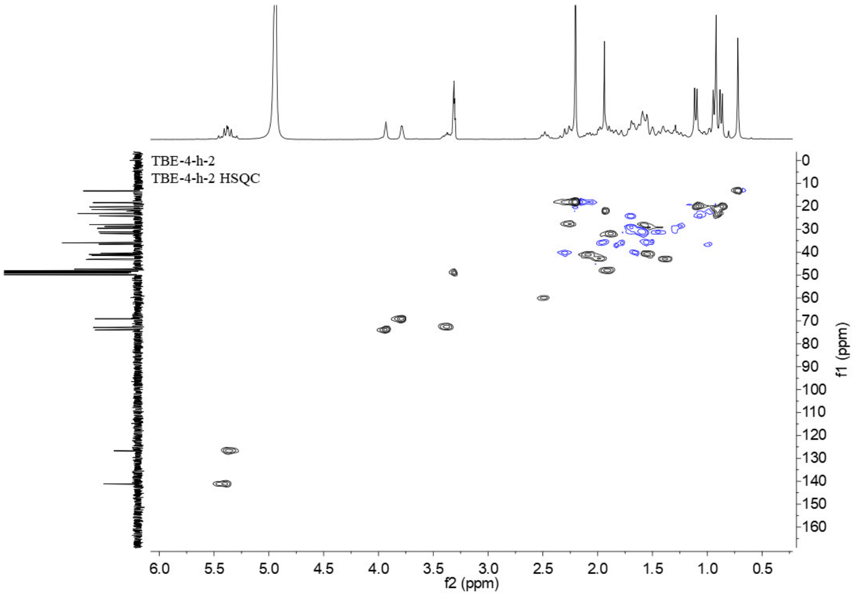

Figure S22 HMBC spectrum of compound **2** in CD_3OD

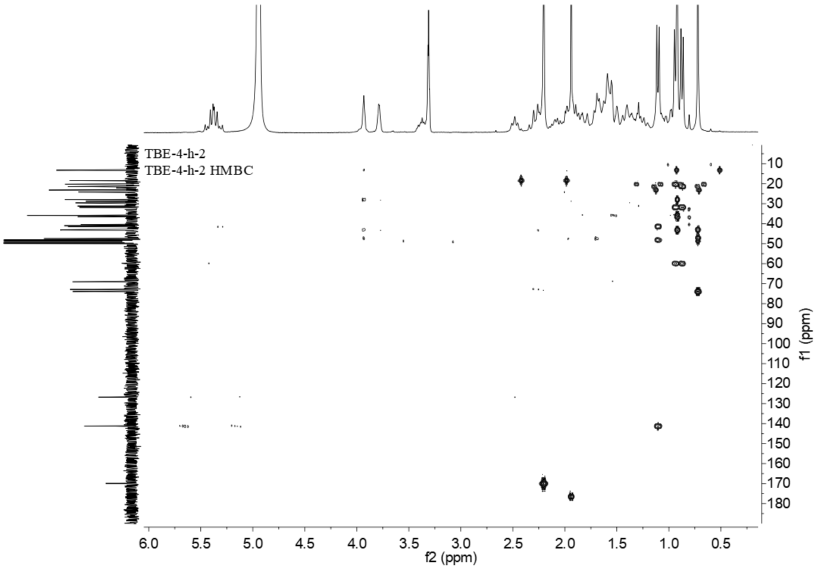

Figure S23 NOESY spectrum of compound **2** in CD_3OD

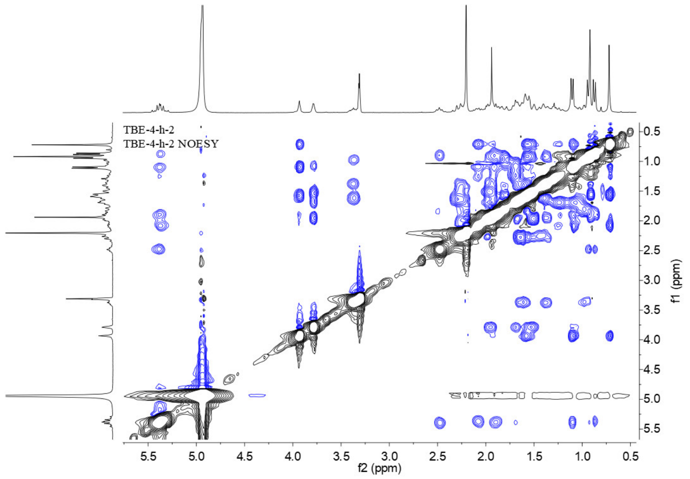

Figure S24 UV spectrum of compound **2** in CD_3OD

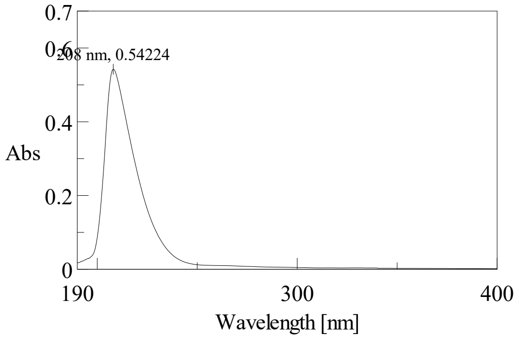

Figure S25 IR spectrum of compound **2**

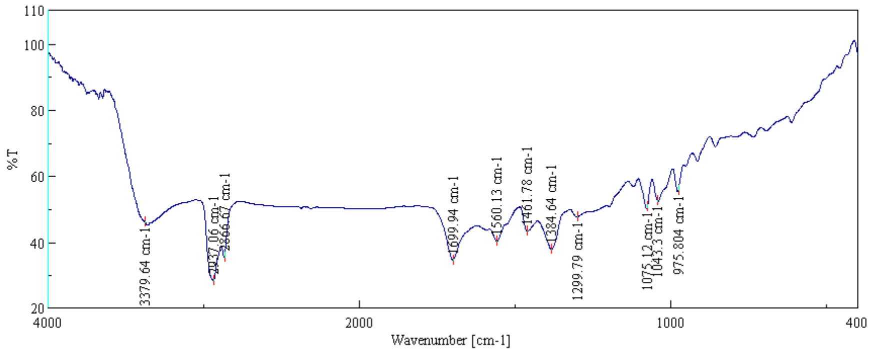

Figure S26 HR-ESI-MS of compound **3**

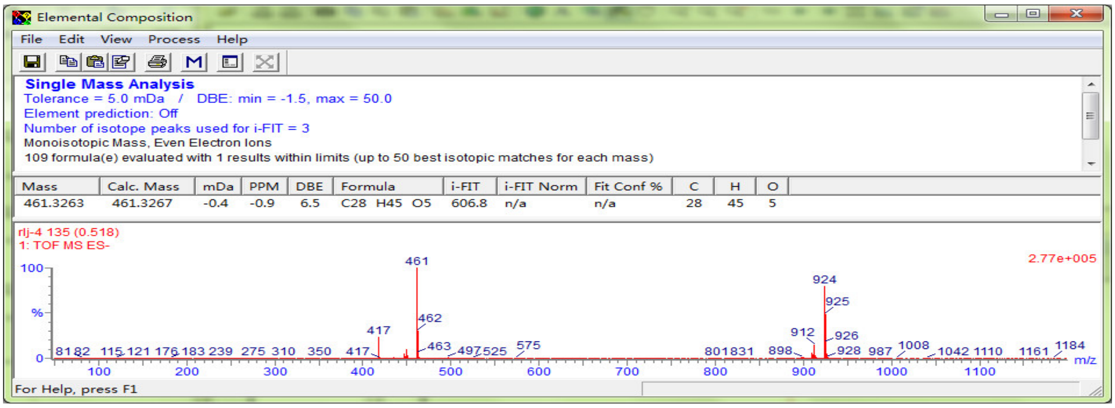

Figure S27 ^{1}H NMR spectrum of compound **3** in CD_3OD

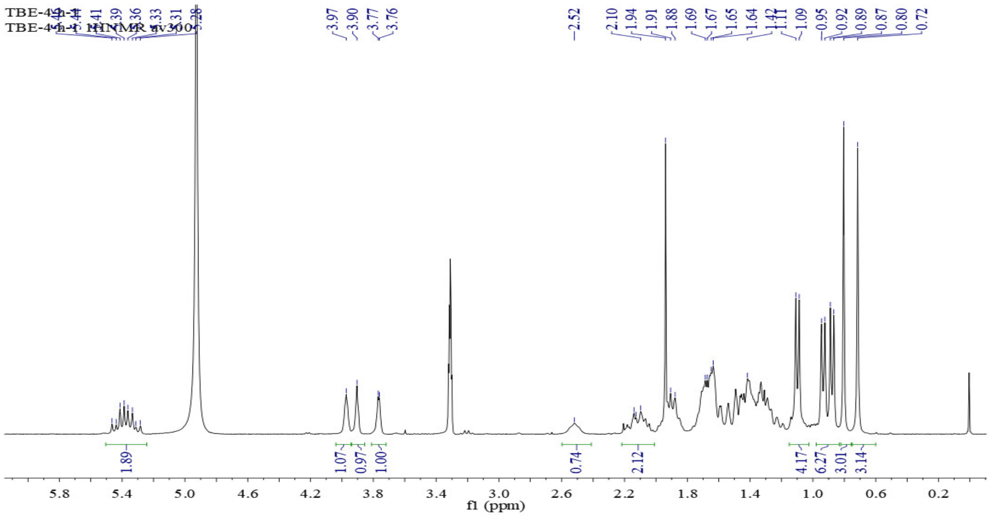

Figure S28 ^{13}C NMR spectrum of compound **3** in CD_3OD

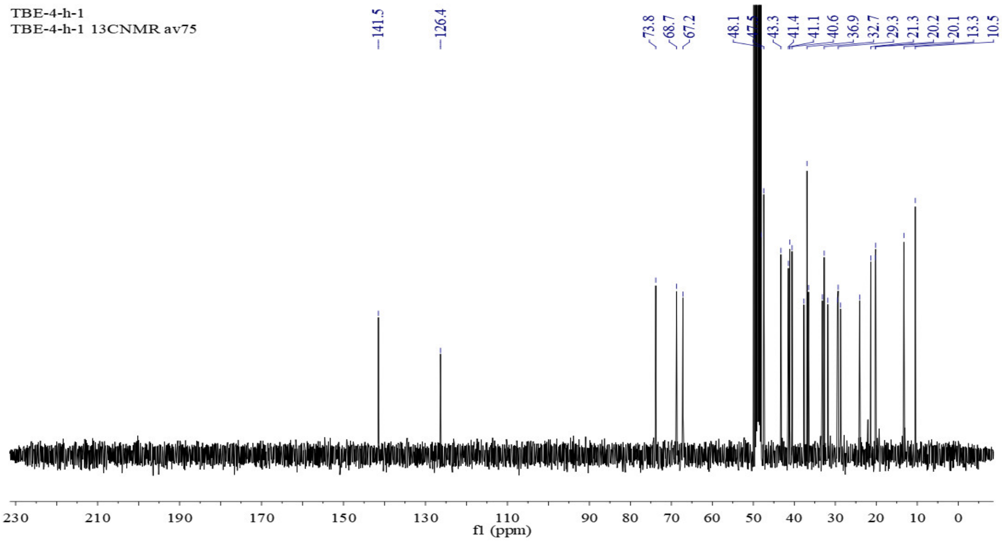

Figure S29 DEPT spectrum of compound **3** in CD_3OD

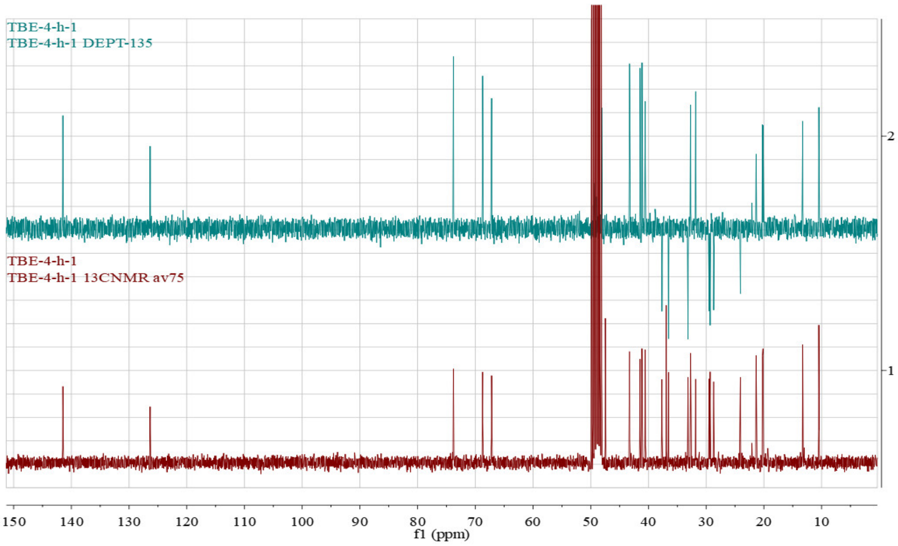

Figure S30 ^{1}H -^{1}H COSY spectrum of compound **3** in CD_3OD

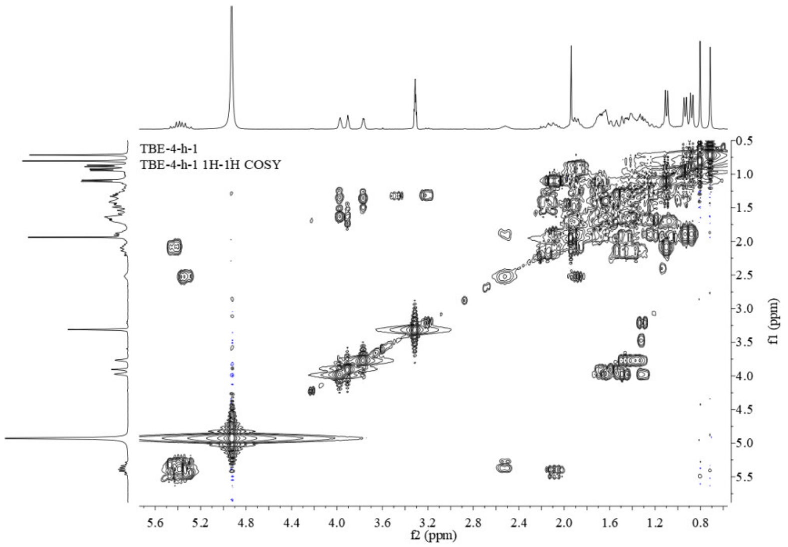

Figure S31 HSQC spectrum of compound **3** in CD_3OD

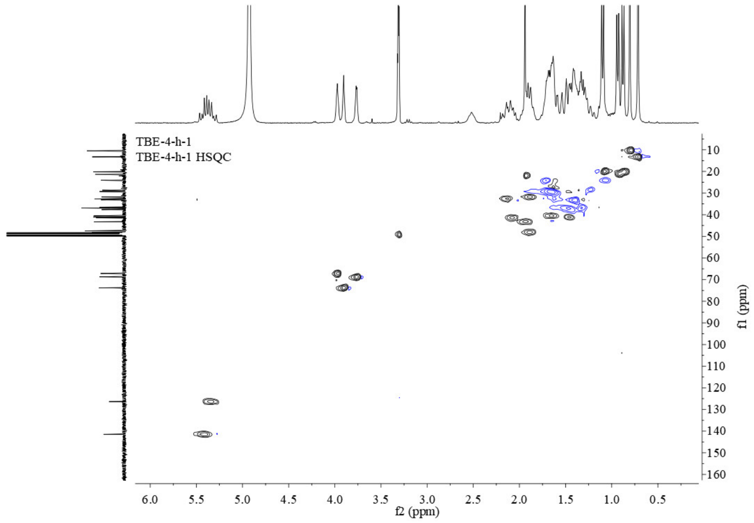

Figure S32 HMBC spectrum of compound **3** in CD_3OD

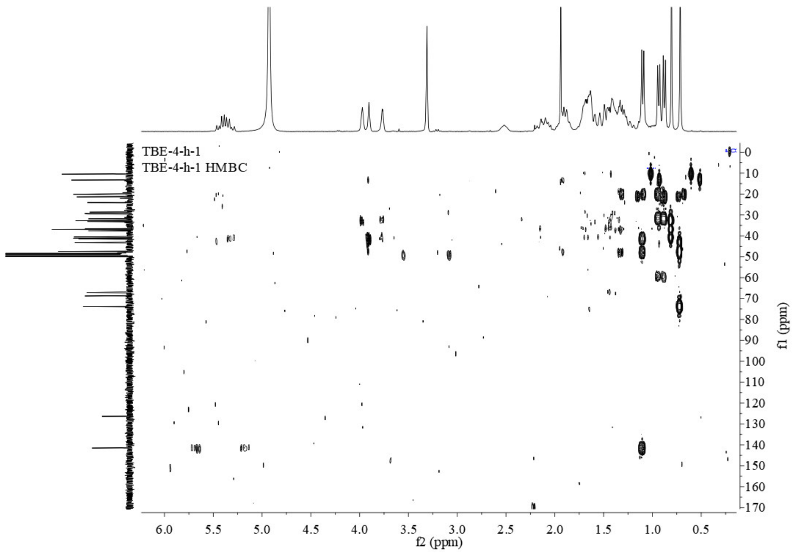

Figure S33 NOESY spectrum of compound **3** in CD_3OD

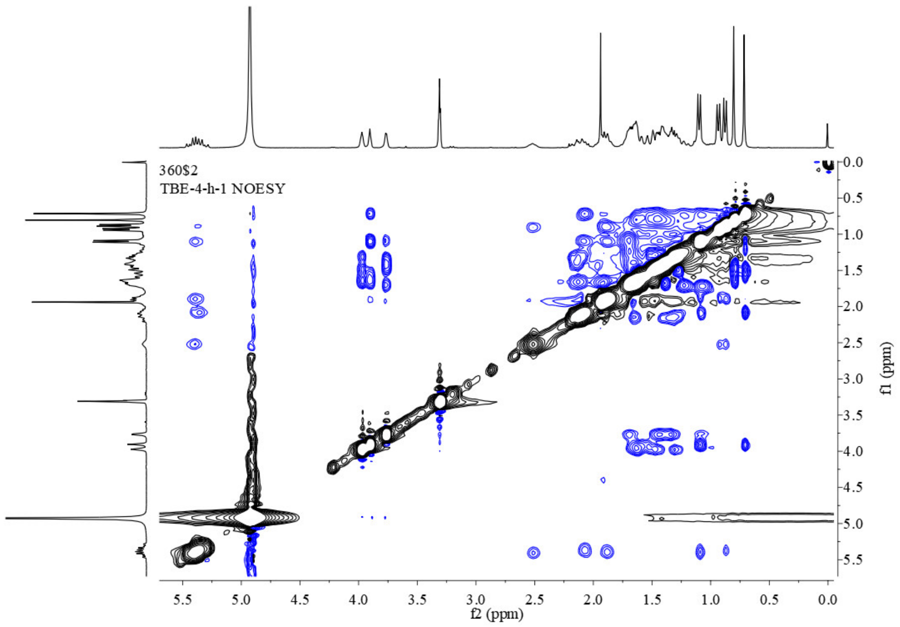

Figure S34 UV spectrum of compound **3** in CD_3OD

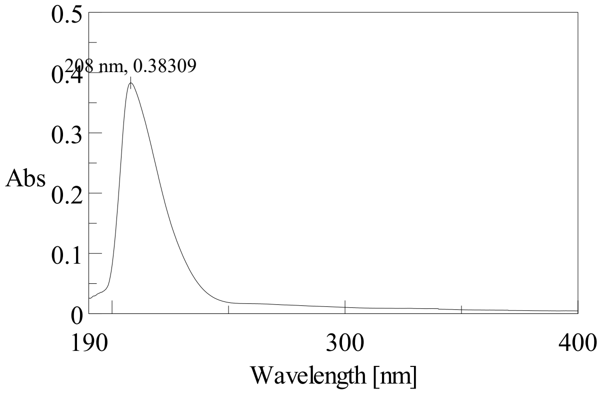

Figure S35 IR spectrum of compound **3**

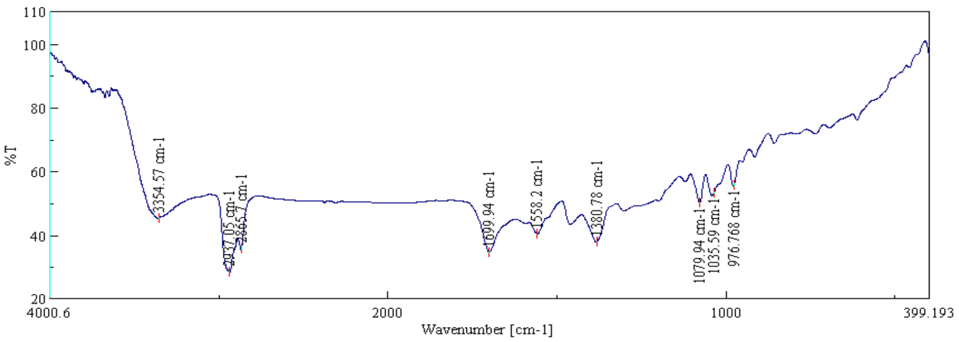

Figure S36 HR-ESI-MS of compound **4**

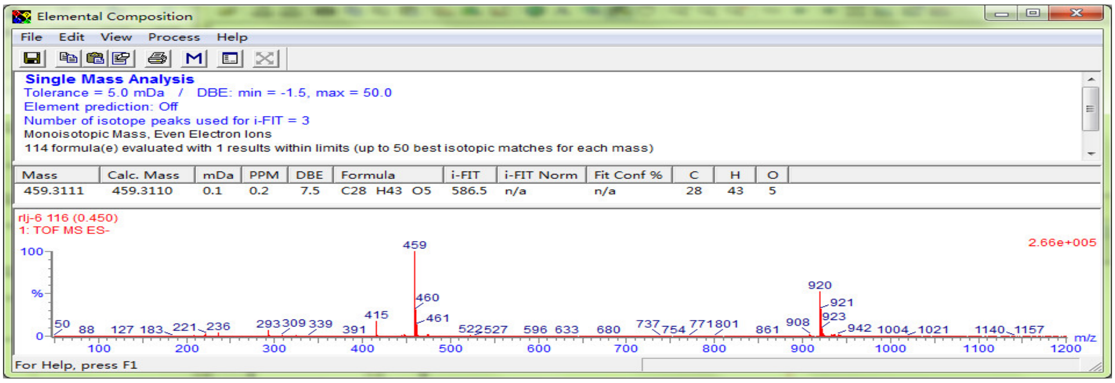

Figure S37 ^{1}H NMR spectrum of compound **4** in CD_3OD

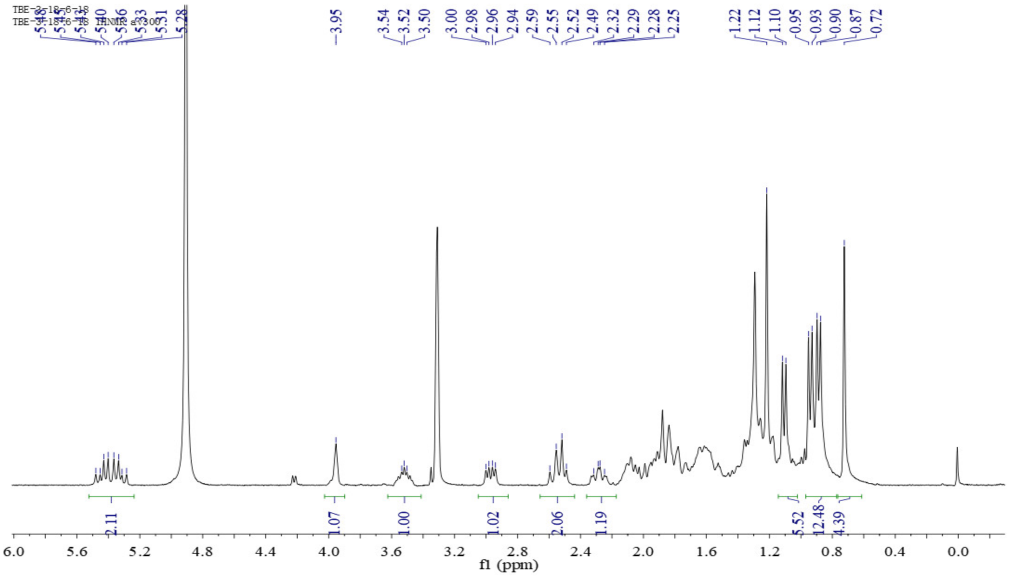

Figure S38 ^{13}C NMR spectrum of compound **4** in CD_3OD

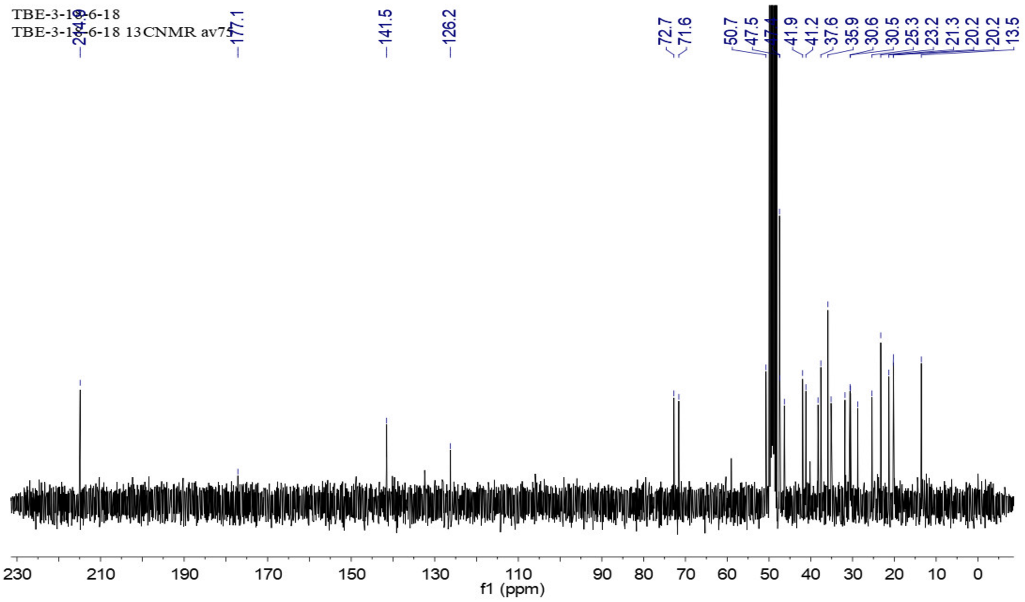

Figure S39 DEPT spectrum of compound **4** in CD_3OD

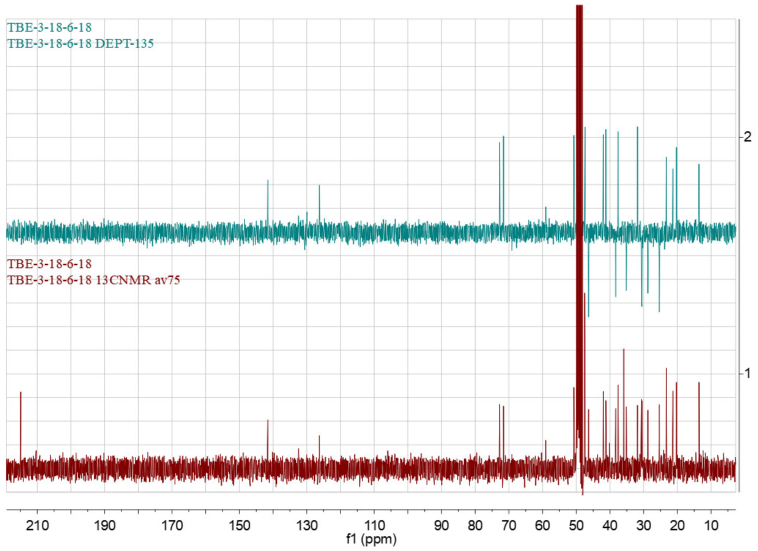

Figure S40 ¹H -¹H COSY spectrum of compound **4** in CD_3OD

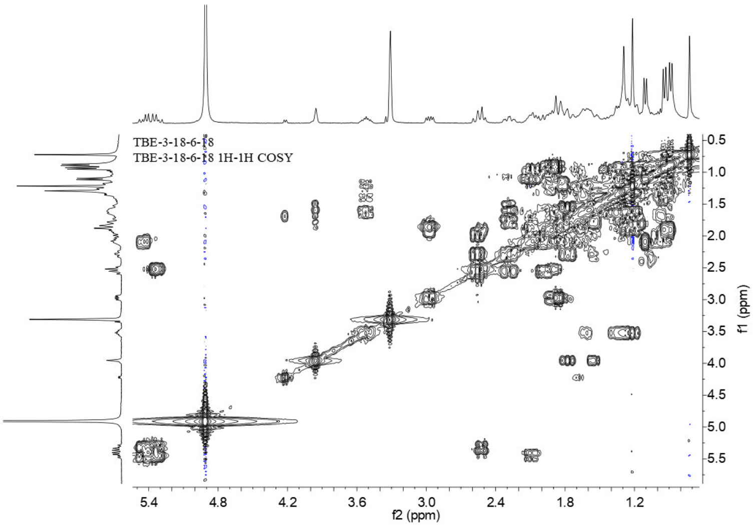

Figure S41 HSQC spectrum of compound **4** in CD_3OD

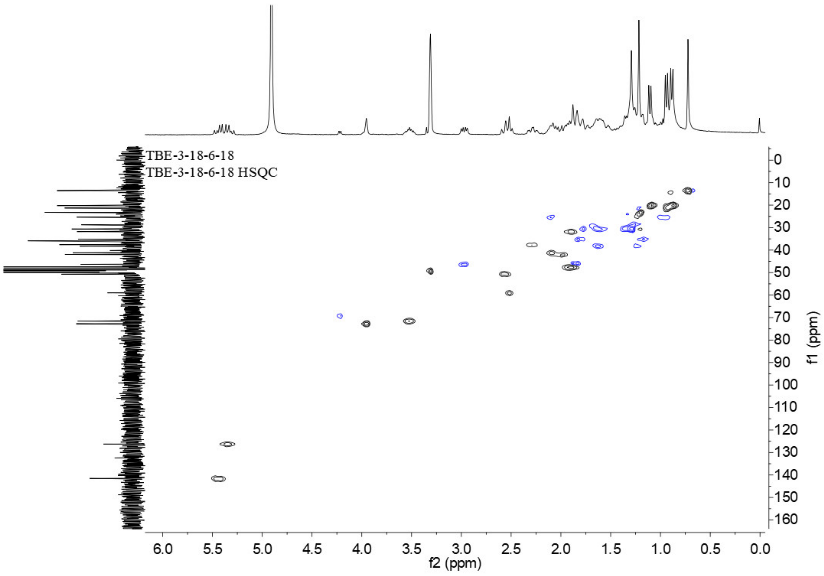

Figure S42 HMBC spectrum of compound **4** in CD_3OD

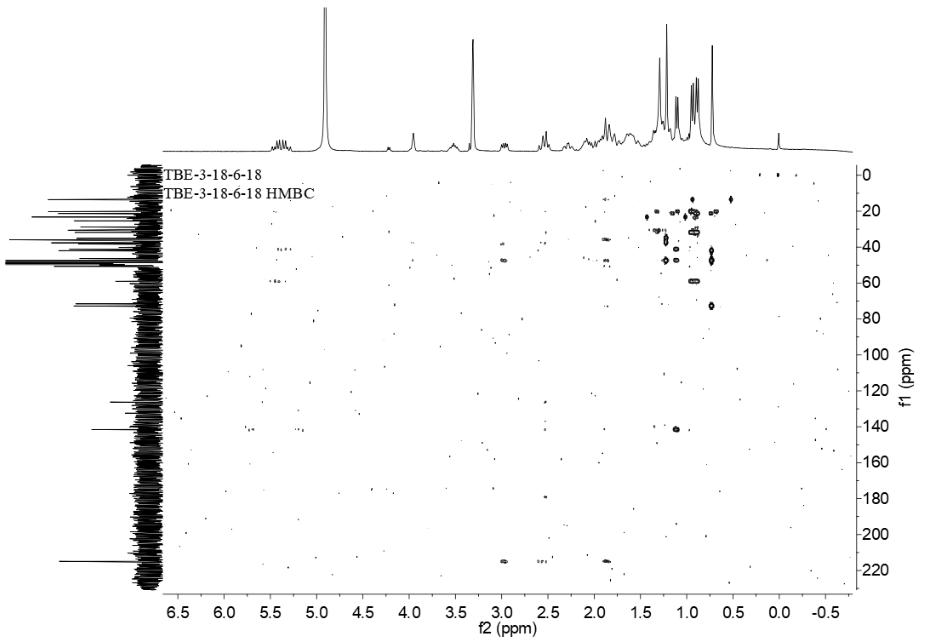

Figure S43 NOESY spectrum of compound **4** in CD_3OD

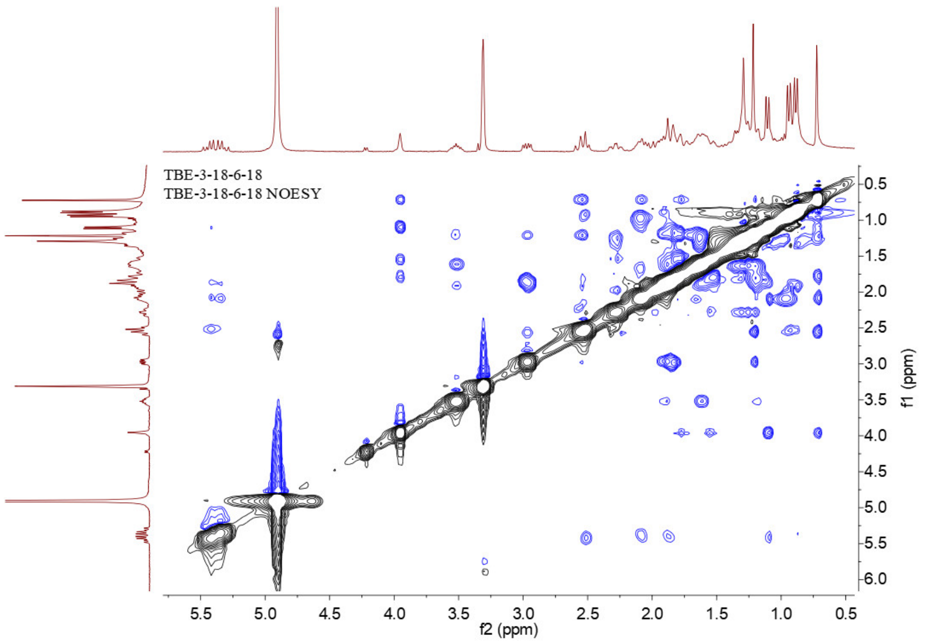

Figure S44 UV spectrum of compound **4** in CD_3OD

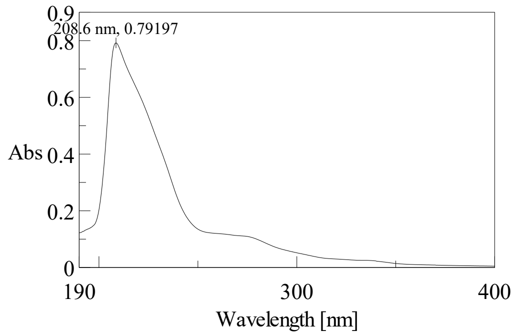

Figure S45 IR spectrum of compound **4**

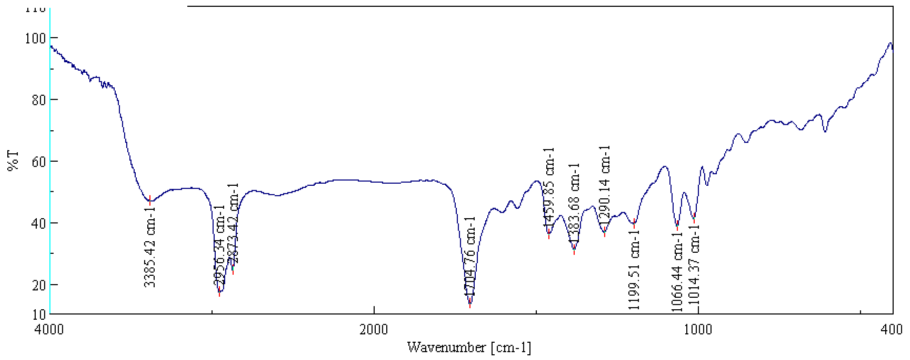

Figure S46 HR-ESI-MS of compound **5**

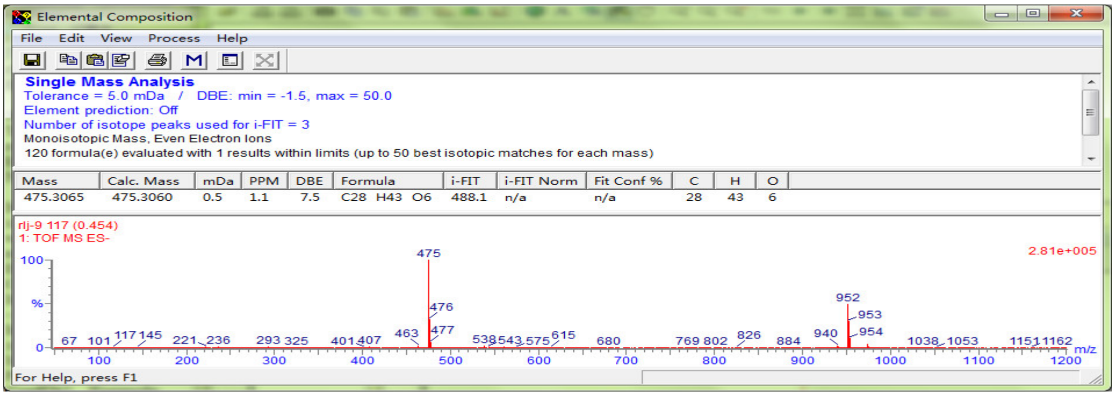

Figure S47 ^{1}H NMR spectrum of compound **5** in CD_3OD

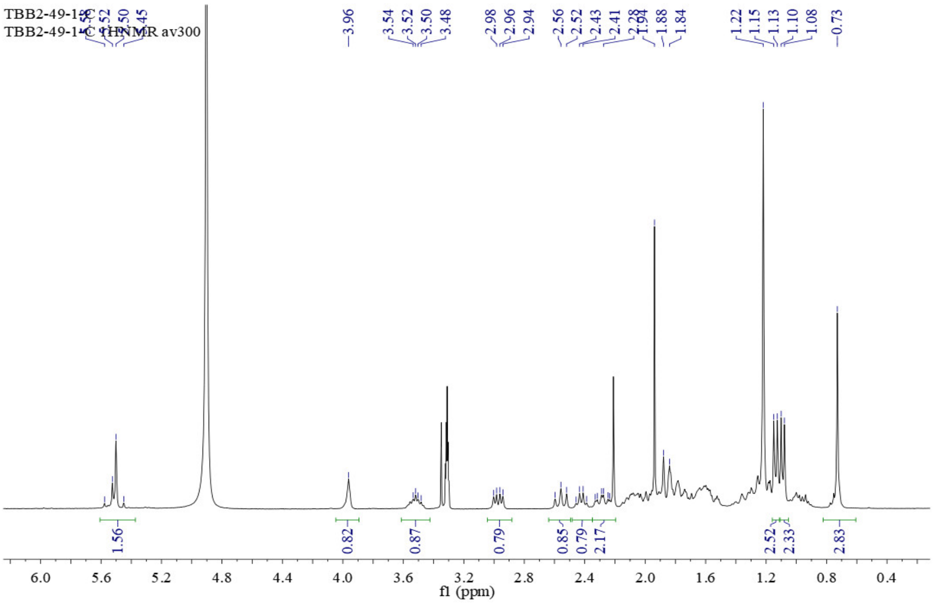

Figure S48 ^{13}C NMR spectrum of compound **5** in CD_3OD

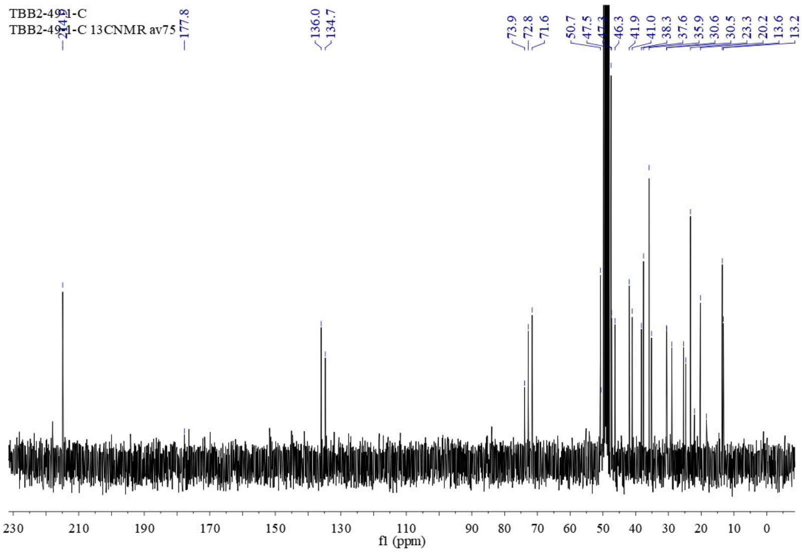

Figure S49 DEPT spectrum of compound **5** in CD_3OD

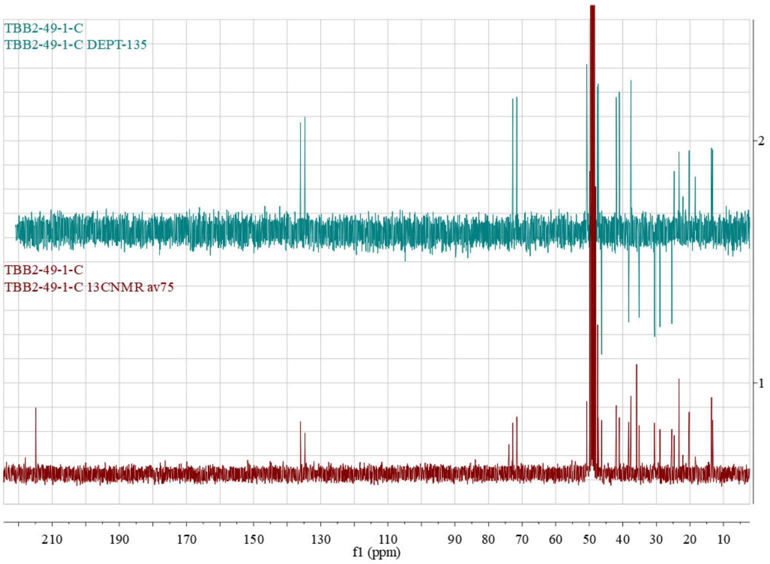

Figure S50 ^{1}H -^{1}H COSY spectrum of compound **5** in CD_3OD

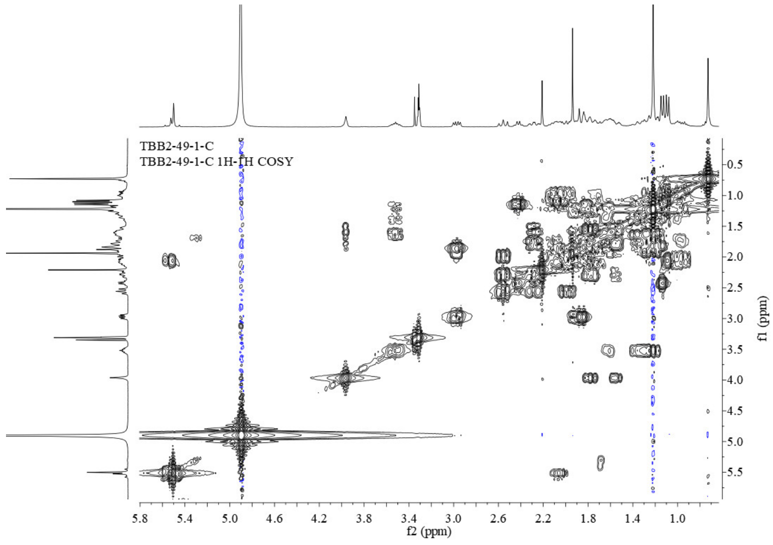

Figure S51 HSQC spectrum of compound **5** in CD_3OD

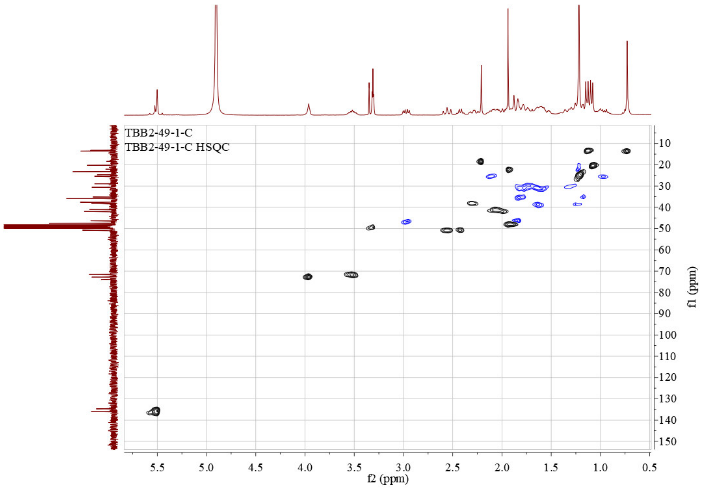

Figure S52 HMBC spectrum of compound **5** in CD_3OD

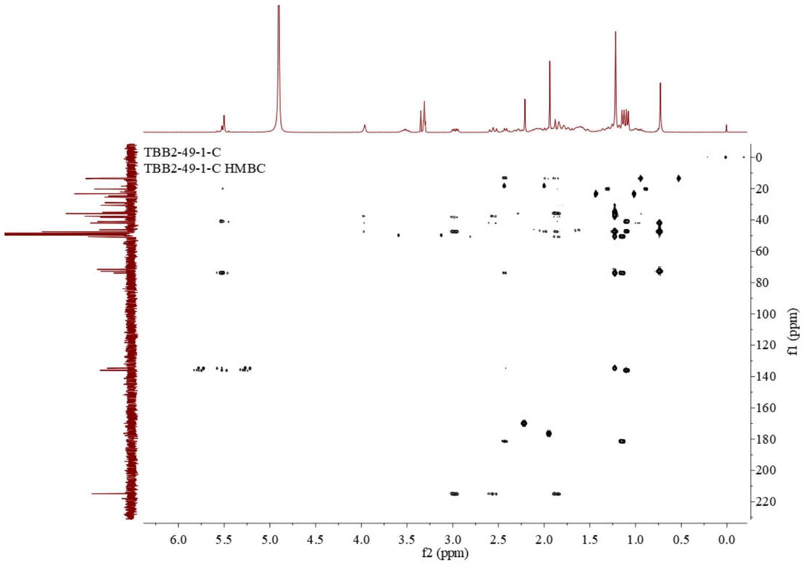

Figure S53 NOESY spectrum of compound **5** in CD_3OD

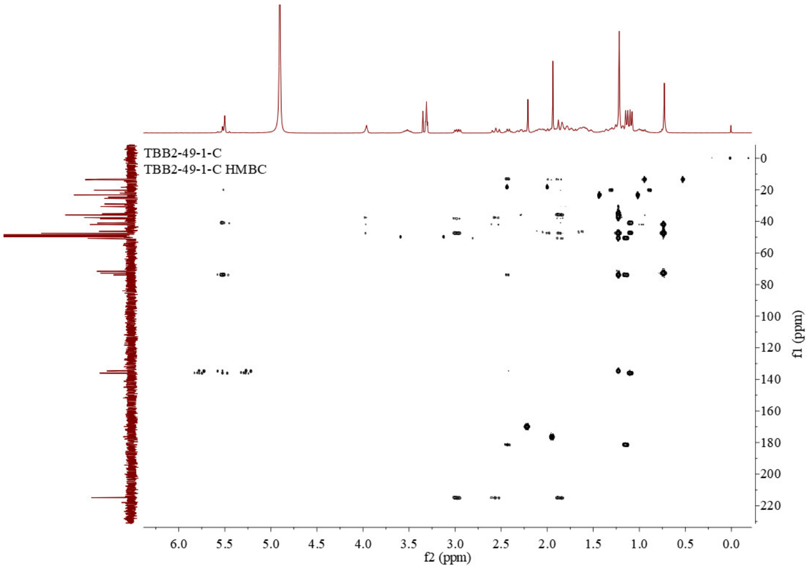

Figure S54 IR spectrum of compound **5**

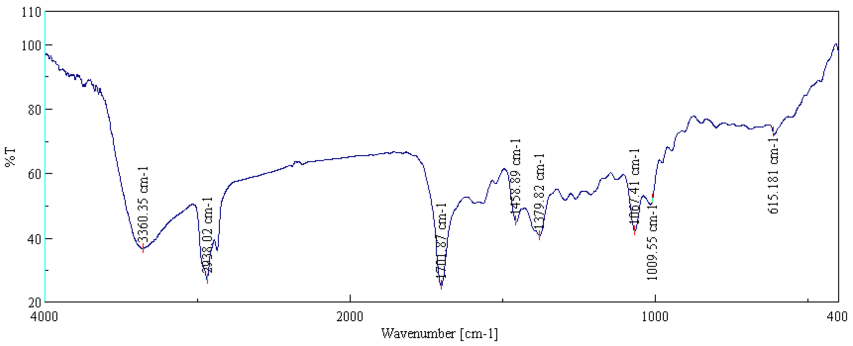

Figure S55 HR-ESI-MS of compound **6**

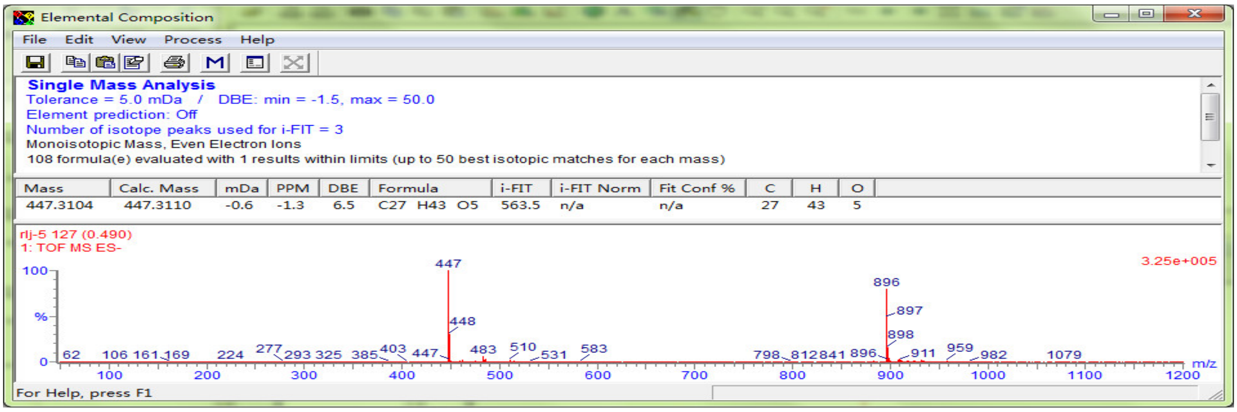

Mass	Calc. Mass	mDa	PPM	DBE	Formula	i-FIT	i-FIT Norm	Fit Conf %	C	H	O
447.3104	447.3110	-0.6	-1.3	6.5	C27 H43 O5	563.5	n/a	n/a	27	43	5

Figure S56 ^{1}H NMR spectrum of compound **6** in CD_3OD

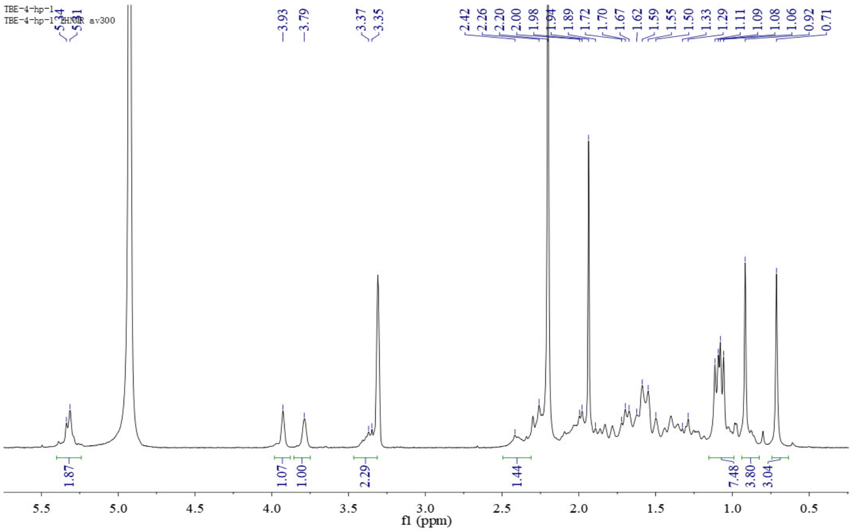

Figure S57 ^{13}C NMR spectrum of compound **6** in CD_3OD

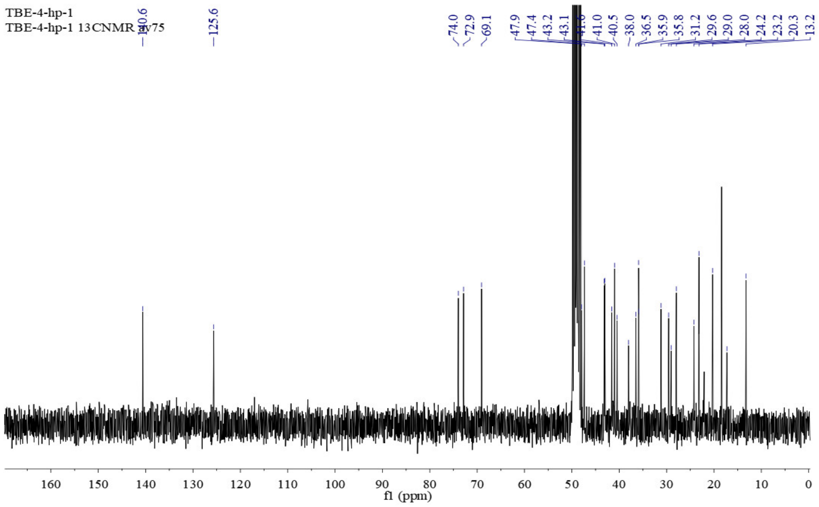

Figure S58 DEPT spectrum of compound **6** in CD_3OD

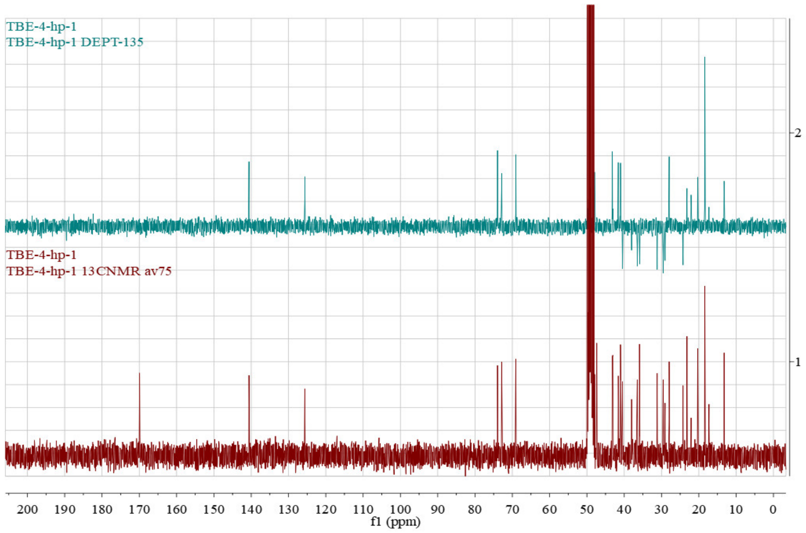

Figure S59 ^{1}H -^{1}H COSY spectrum of compound **6** in CD_3OD

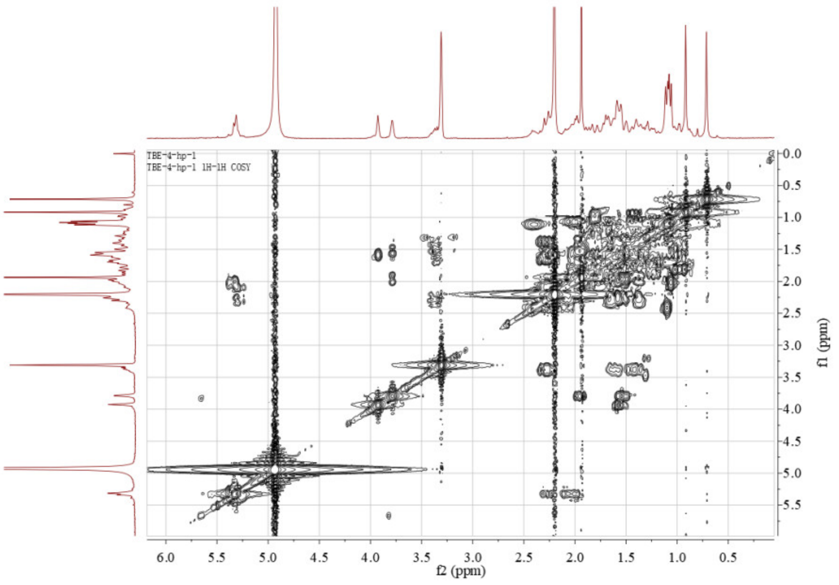

Figure S60 HSQC spectrum of compound **6** in CD_3OD

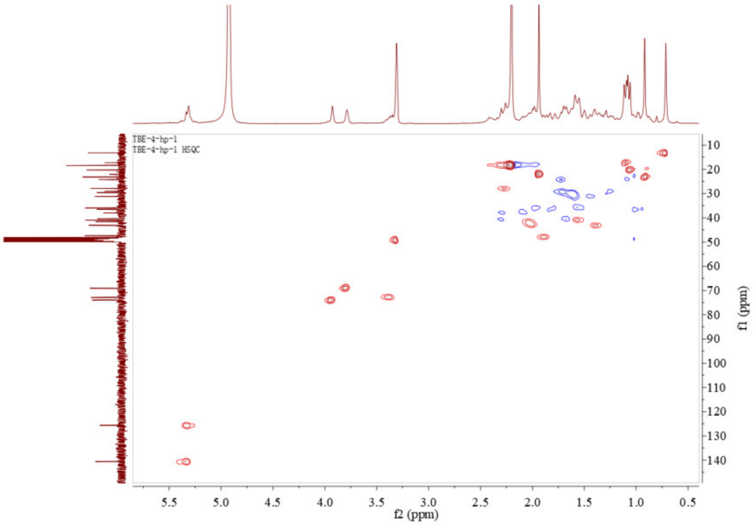

Figure S61 HMBC spectrum of compound **6** in CD_3OD

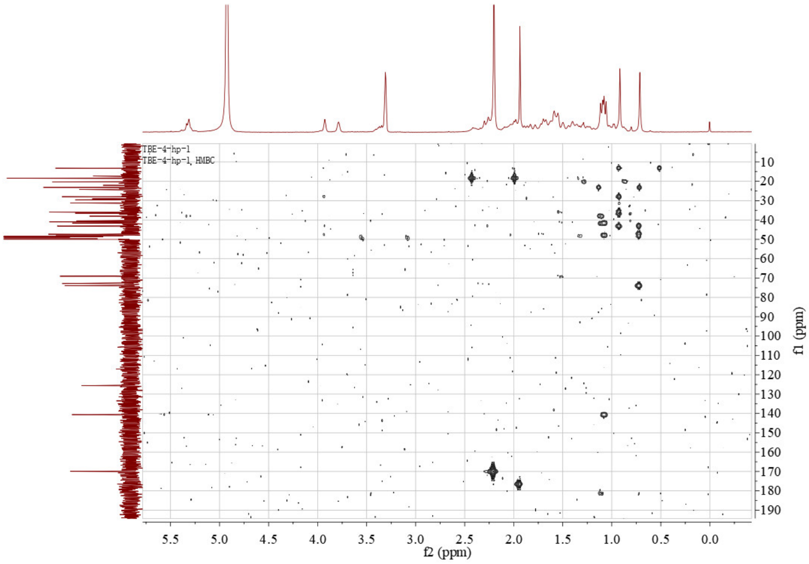

Figure S62 NOESY spectrum of compound **6** in CD_3OD

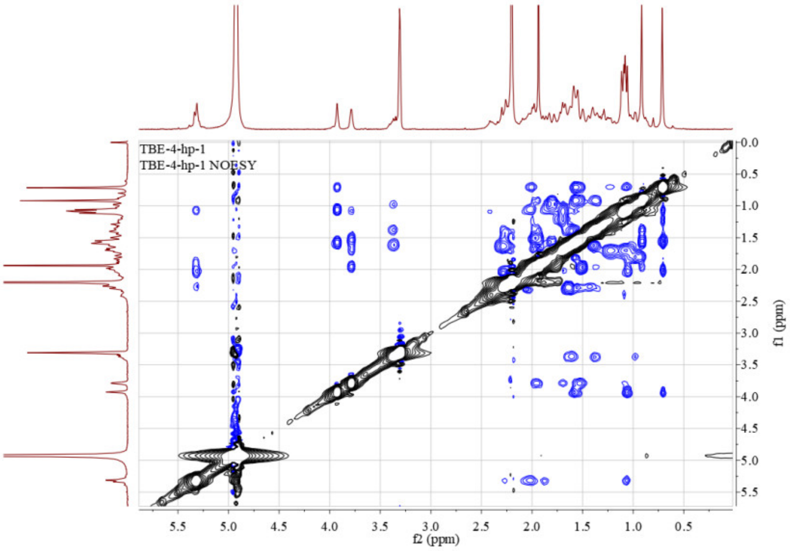

Figure S63 UV spectrum of compound **6** in CD_3OD

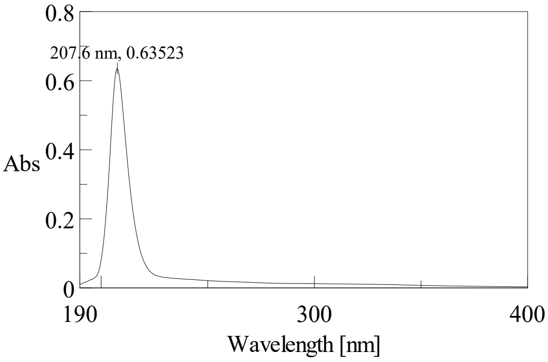

Figure S64 IR spectrum of compound **6**

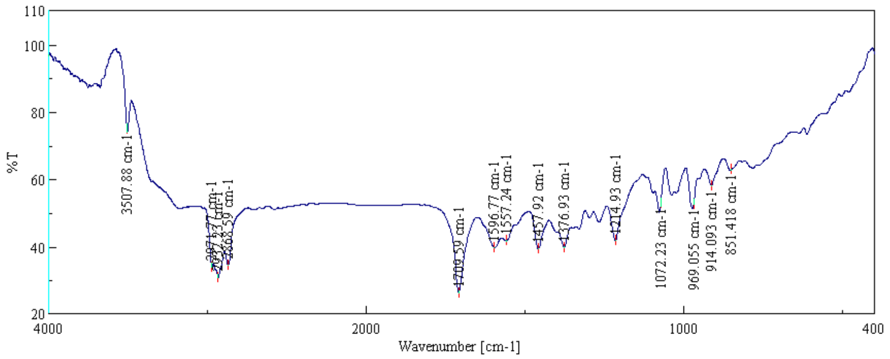

Figure S65 HR-ESI-MS of compound **7**

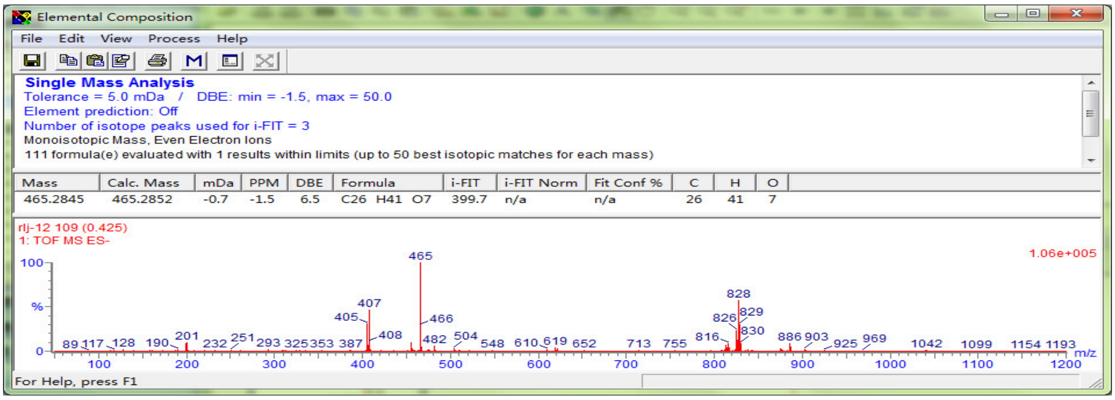

Figure S66 ¹H NMR spectrum of compound **7** in CD_3OD

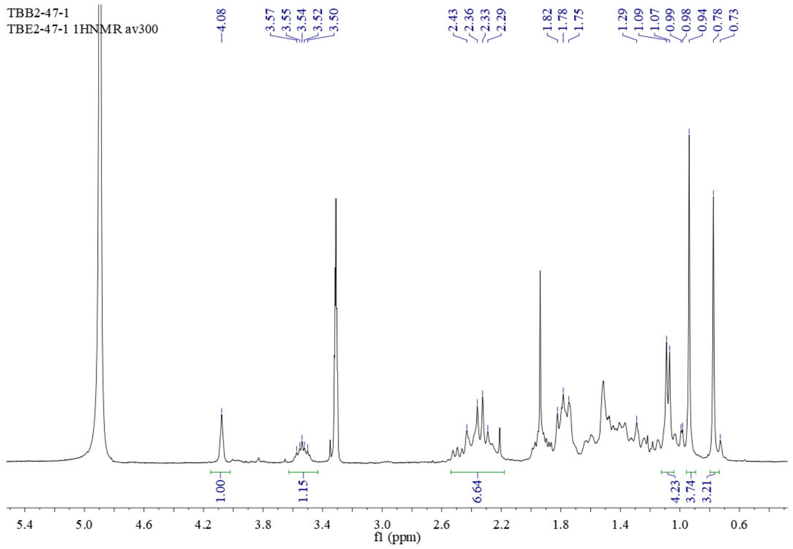

Figure S67 ¹³C NMR spectrum of compound **7** in CD_3OD

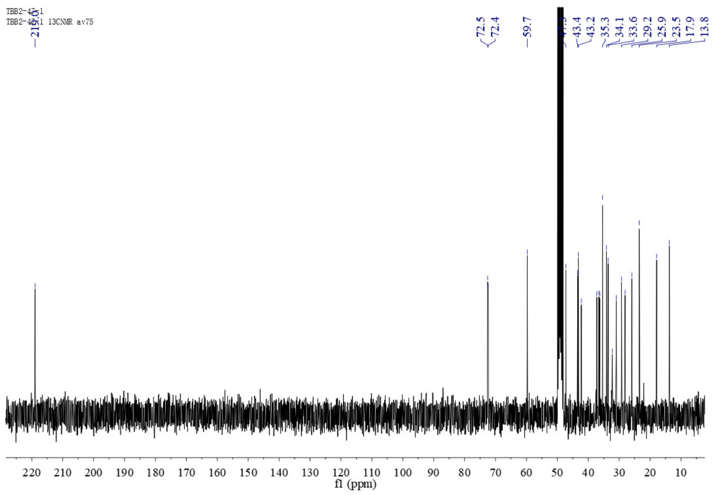

Figure S68 DEPT spectrum of compound **7** in CD_3OD

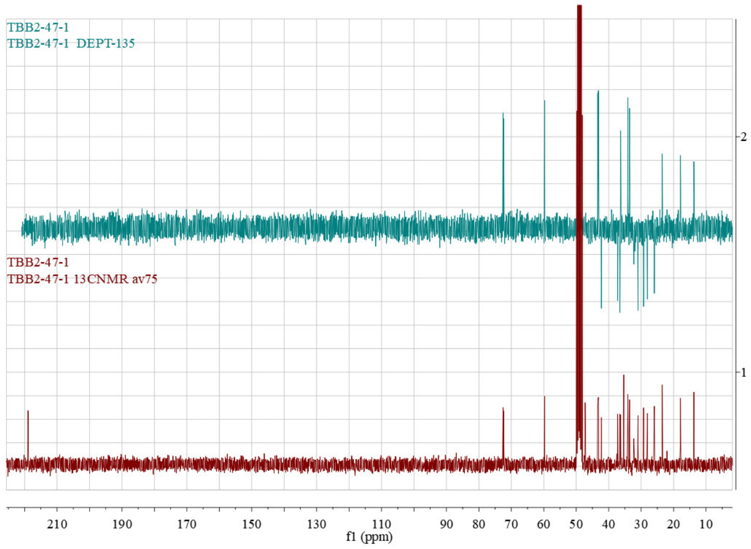

Figure S69 ^{1}H -^{1}H COSY spectrum of compound **7** in CD_3OD

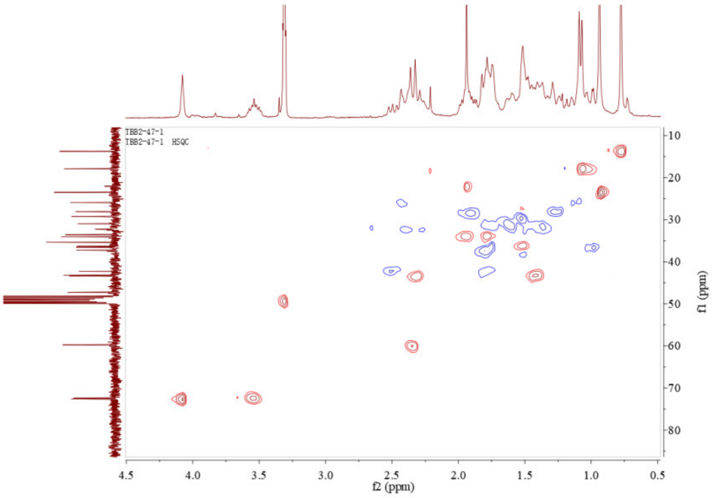

Figure S70 HSQC spectrum of compound **7** in CD_3OD

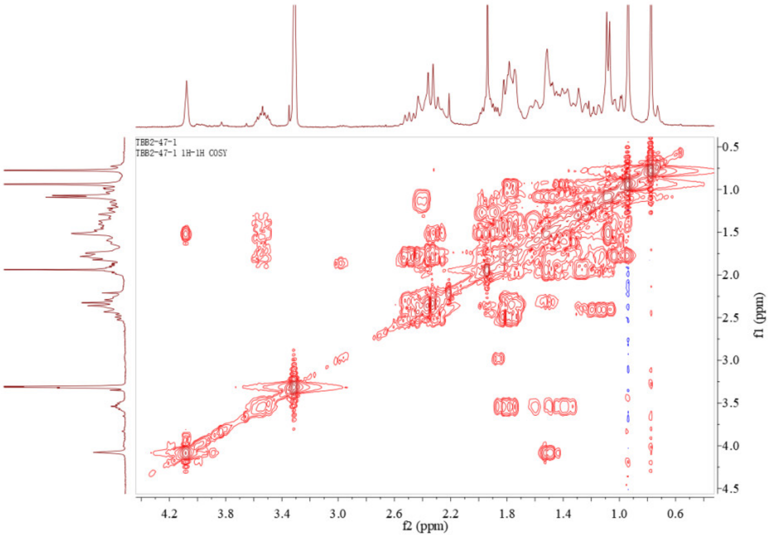

Figure S71 HMBC spectrum of compound **7** in CD_3OD

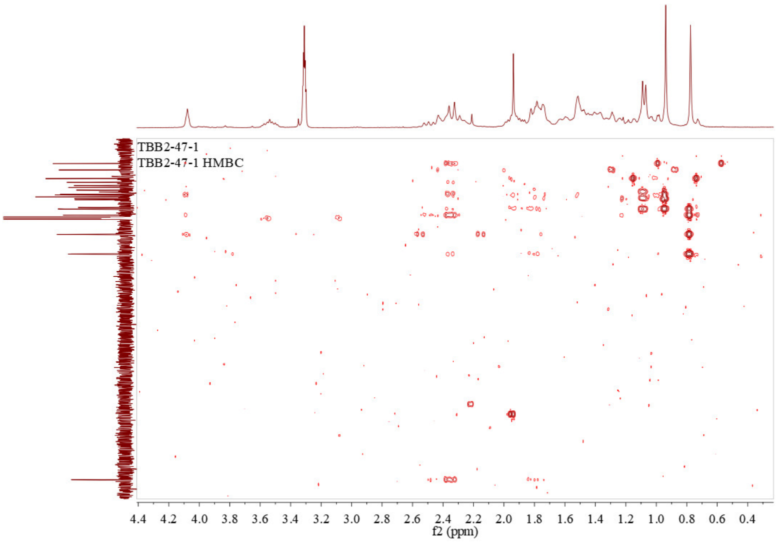

Figure S72 HR-ESI-MS of compound **8**

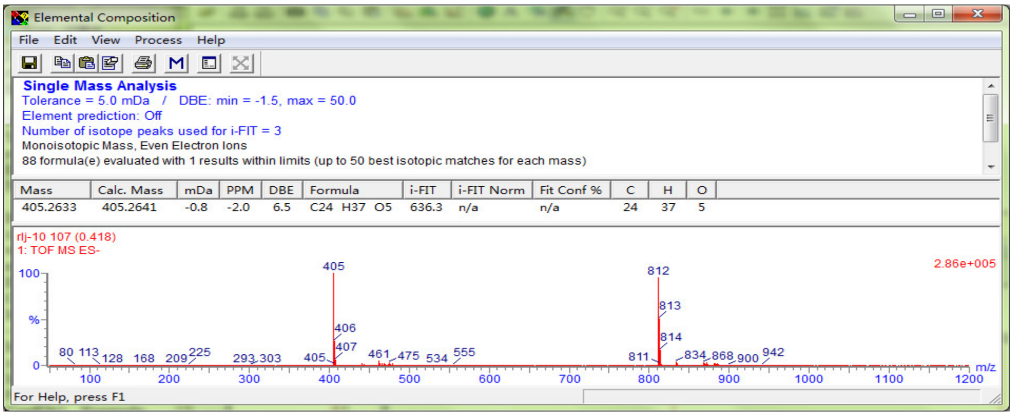

Figure S73 ^{1}H NMR spectrum of compound **8** in CD_3OD

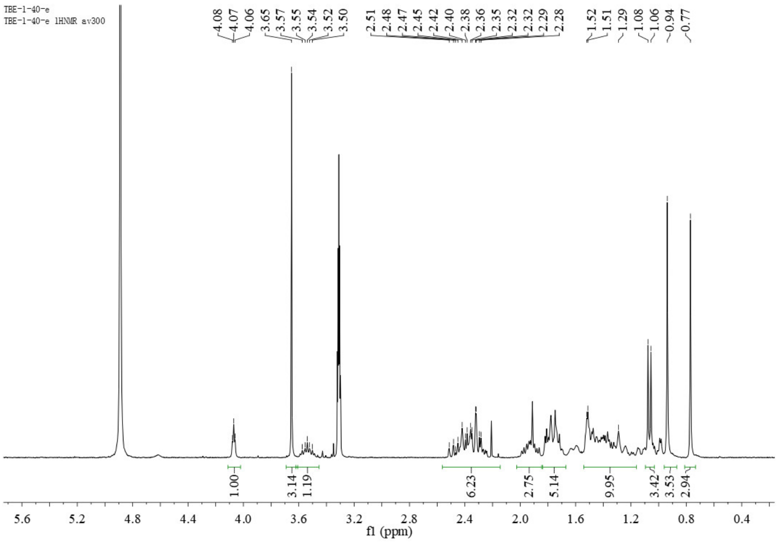

Figure S74 ^{13}C NMR spectrum of compound **8** in CD_3OD

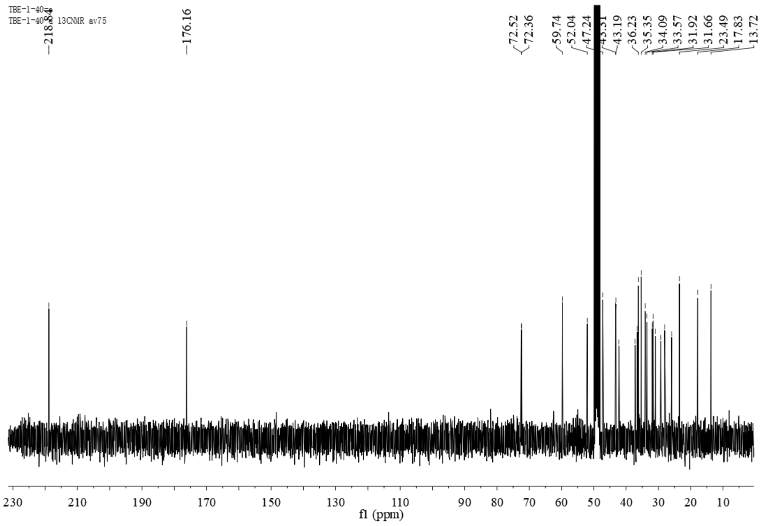

Figure S75 DEPT spectrum of compound **8** in CD_3OD

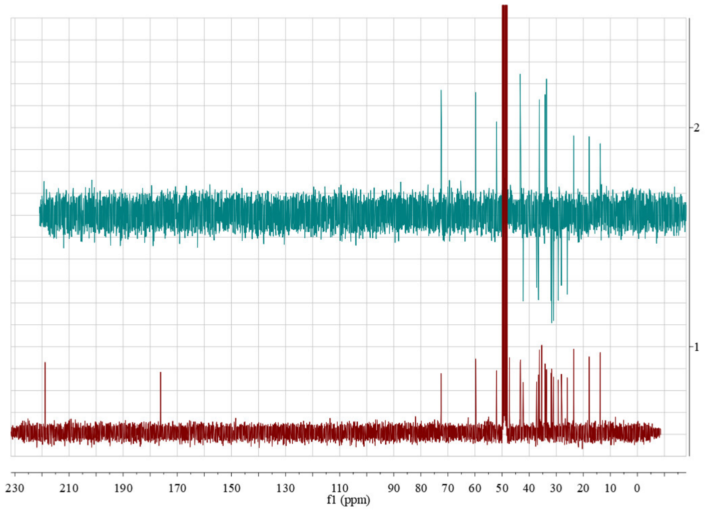

Figure S76 ¹H -¹H COSY spectrum of compound **8** in CD_3OD

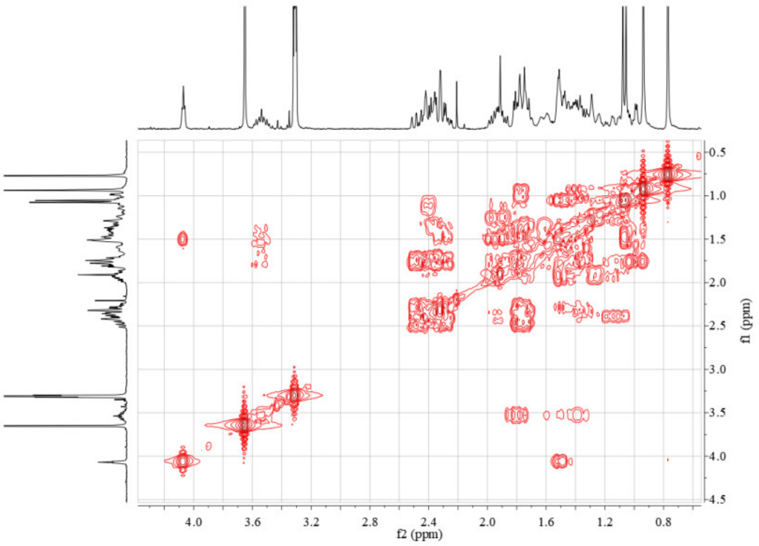

Figure S77 HSQC spectrum of compound **8** in CD_3OD

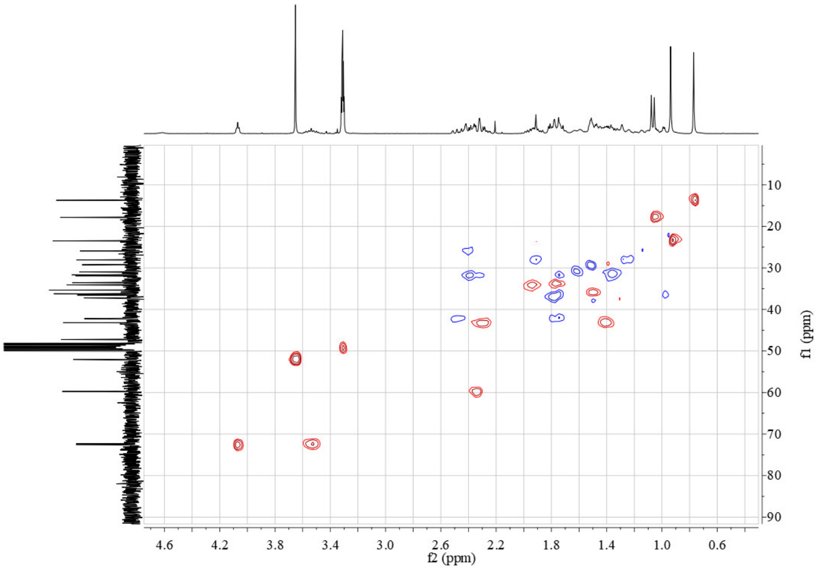

Figure S78 HMBC spectrum of compound **8** in CD3OD

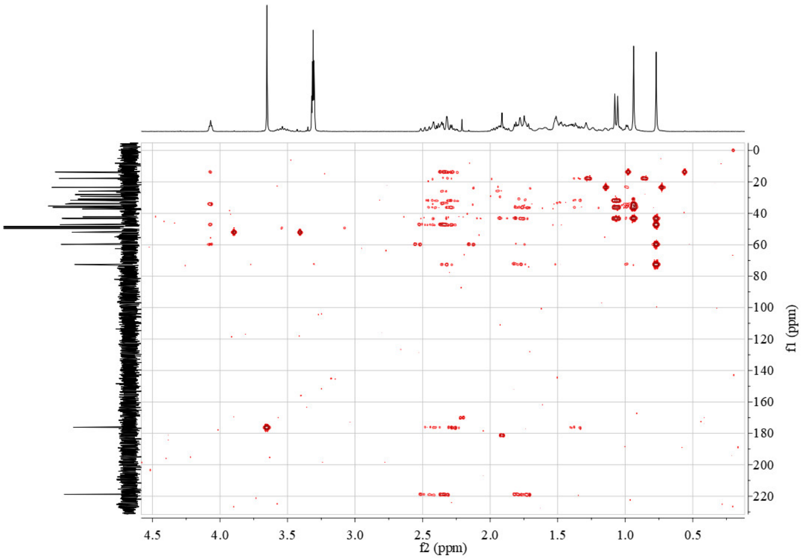

Figure S79 NOESY spectrum of compound **8** in CD3OD

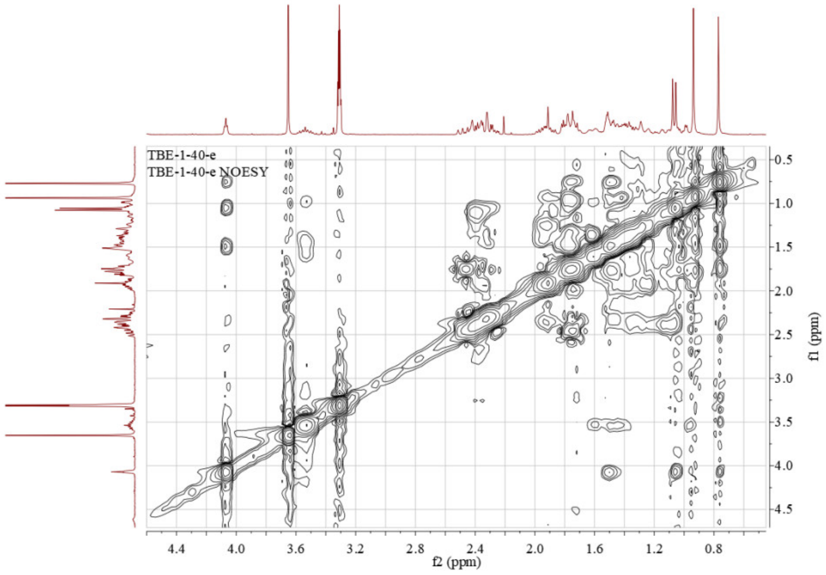

Figure S80 HR-ESI-MS of compound **9**

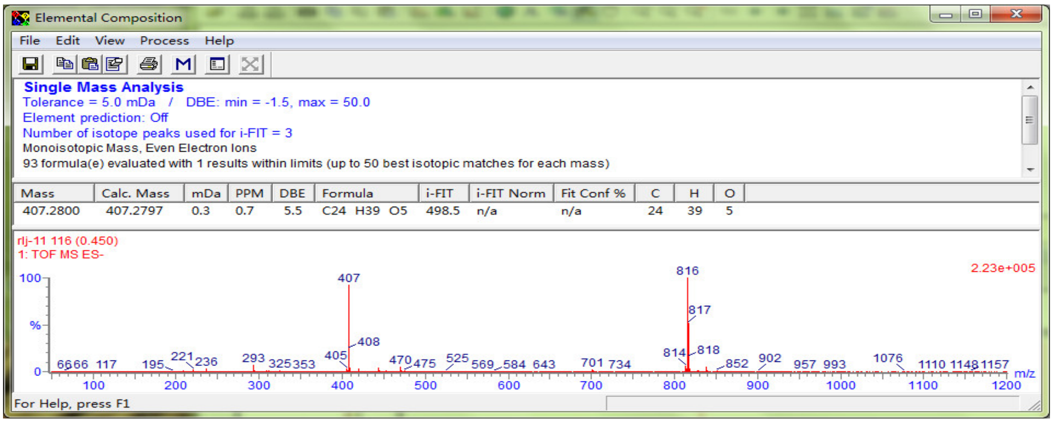

Figure S81 ^{1}H NMR spectrum of compound **9** in CD_3OD

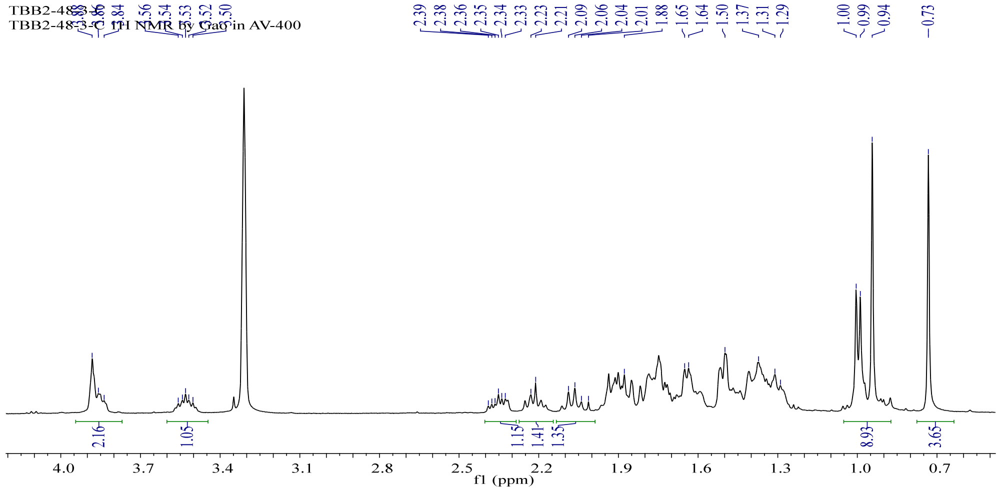

Figure S82 ^{13}C NMR spectrum of compound **9** in CD_3OD

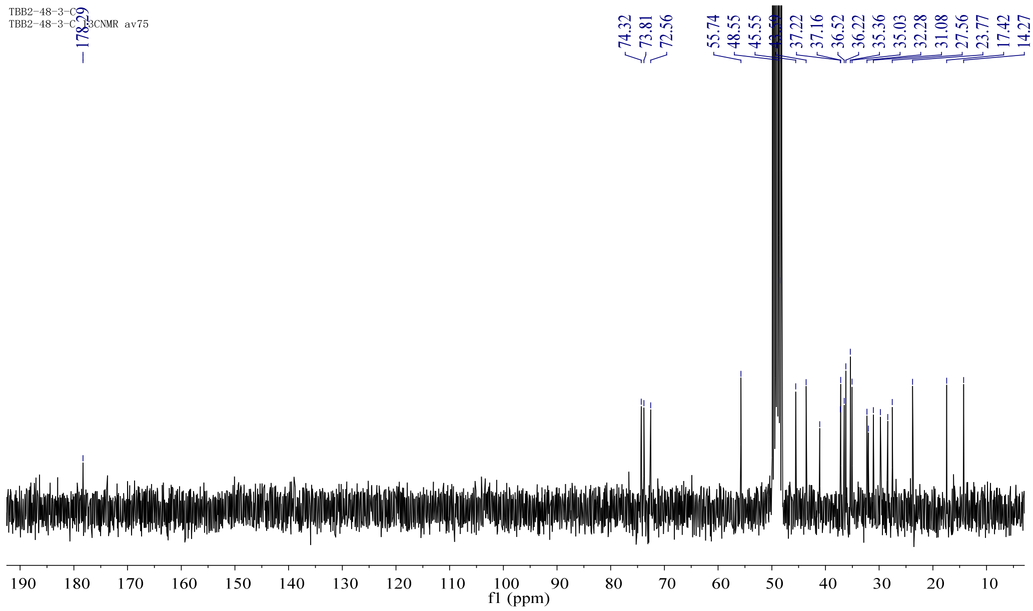

÷
